# New Cytotoxic Natural Products from the Red Sea Sponge *Stylissa carteri*

**DOI:** 10.3390/md18050241

**Published:** 2020-05-03

**Authors:** Reda F. A. Abdelhameed, Eman S. Habib, Nermeen A. Eltahawy, Hashim A. Hassanean, Amany K. Ibrahim, Anber F. Mohammed, Shaimaa Fayez, Alaa M. Hayallah, Koji Yamada, Fathy A. Behery, Mohammad M. Al-Sanea, Sami I. Alzarea, Gerhard Bringmann, Safwat A. Ahmed, Usama Ramadan Abdelmohsen

**Affiliations:** 1 Department of Pharmacognosy, Faculty of Pharmacy, Suez Canal University, Ismailia 41522, Egypt; omarreda_70@yahoo.com (R.F.A.A.); emy_197@hotmail.com (E.S.H.); nermeenazmy25@gmail.com (N.A.E.); hasanean2000@yahoo.com (H.A.H.); am_kamal66@yahoo.com (A.K.I.); 2 Department of Pharmaceutical Organic Chemistry, Faculty of Pharmacy, Assiut University, Assiut 71526, Egypt; anber_pharm_2006@yahoo.com (A.F.M.); alaa_hayalah@yahoo.com (A.M.H.); 3 Institute of Organic Chemistry, University of Würzburg, Am Hubland, 97074 Würzburg, Germany; shaimaa.seaf@uni-wuerzburg.de (S.F.); bringmann@chemie.uni-wuerzburg.de (G.B.); 4 Department of Pharmacognosy, Faculty of Pharmacy, Ain-Shams University, Cairo 11566, Egypt; 5 Department of Pharmaceutical Chemistry, Faculty of Pharmacy, Deraya University, New Minia 61111, Egypt; 6 Graduate School of Biomedical Sciences, Nagasaki University, Bunkyo-machi 1-14, Nagasaki 852-8521, Japan; kyamada@nagasaki-u.ac.jp; 7 Department of Pharmacognosy, Faculty of Pharmacy, Mansoura University, Mansoura 35516, Egypt; fathybehery@yahoo.com; 8 Department of Pharmaceutical Sciences, College of Pharmacy, Riyadh Elm University, Riyadh 11681, Saudi Arabia; 9 Department of Pharmaceutical Chemistry, College of Pharmacy, Jouf University, Aljouf 72341, Saudi Arabia; mohmah80@gmail.com; 10 Department of Pharmacology, College of Pharmacy, Jouf University, Aljouf 72341, Saudi Arabia; samisz@ju.edu.sa; 11 Department of Pharmacognosy, Faculty of Pharmacy, Deraya University, New Minia 61111, Egypt; 12 Department of Pharmacognosy, Faculty of Pharmacy, Minia University, Minia 61519, Egypt

**Keywords:** LC-HRESIMS, *Stylissa carteri*, ceramide, cerebroside, docking, cytotoxic activity

## Abstract

Bioactivity-guided isolation supported by LC-HRESIMS metabolic profiling led to the isolation of two new compounds, a ceramide, stylissamide A (**1**), and a cerebroside, stylissoside A (**2**), from the methanol extract of the Red Sea sponge *Stylissa carteri*. Structure elucidation was achieved using spectroscopic techniques, including 1D and 2D NMR and HRMS. The bioactive extract’s metabolomic profiling showed the existence of various secondary metabolites, mainly oleanane-type saponins, phenolic diterpenes, and lupane triterpenes. The in vitro cytotoxic activity of the isolated compounds was tested against two human cancer cell lines, MCF-7 and HepG2. Both compounds, **1** and **2**, displayed strong cytotoxicity against the MCF-7 cell line, with IC_50_ values at 21.1 ± 0.17 µM and 27.5 ± 0.18 µM, respectively. They likewise showed a promising activity against HepG2 with IC_50_ at 36.8 ± 0.16 µM for **1** and IC_50_ 30.5 ± 0.23 µM for **2** compared to the standard drug cisplatin. Molecular docking experiments showed that **1** and **2** displayed high affinity to the SET protein and to inhibitor 2 of protein phosphatase 2A (I2PP2A), which could be a possible mechanism for their cytotoxic activity. This paper spreads light on the role of these metabolites in holding fouling organisms away from the outer surface of the sponge, and the potential use of these defensive molecules in the production of novel anticancer agents.

## 1. Introduction

Marine environments have proven to be an important source of unique chemical entities with a wide range of biological activities [1,2,3,4,5,6]. Owing to its biodiversity and seasonal variations in air and water temperatures, the Red Sea is one of the most important areas for marine research. Marine sponges are soft-bodied, sessile organisms which belong to the Porifera phylum. In both salt and freshwater ecosystems, more than 8000 species of sponges have been described. The sessile nature of marine sponges has led to the development of mechanisms for chemical defense to deter marine predators such as sharks, tortoises, and invertebrates [7]. Marine sponges are therefore an extremely rich source of secondary metabolites possessing various biological activities. Investigation of Red Sea marine sponges permitted detection of a wide range of secondary metabolites, including terpenes, alkaloids, sterols, steroidal glycosides, and ceramides [8,9,10]. Ceramides are bioactive lipids, which have been found in many marine invertebrate organisms. These compounds are involved in a number of physiological functions including apoptosis, arrest of cell growth, and cell-senescence [11]. They have also been reported to be precursors of complex sphingolipids (SLs). Ceramides possessing cytotoxic activity have previously been isolated from marine sponges [12]. The marine sponge *Stylissa carteri* is abundant in coastal Red Sea reefs, typically at depths between 5 and 15 m. Numerous secondary metabolites have already been isolated from *S. carteri*, including alkaloids [13], cyclic heptapeptides [14], and the pyrrole-2-aminoimidazoles stylissazoles A-C [15]. Alkaloids isolated from *S. carteri* were suggested to be prospective supports for human immunodeficiency virus (HIV) inhibition [16]. In addition, *Stylissa carteri*-associated bacteria were reported to have anti-plasmodial activity [17]. In this study, a bioactivity-guided fractionation was performed assisted by LC-HRESIMS investigation of the Red Sea sponge *Stylissa carteri* methanolic extract, which led to the isolation of two new compounds, stylissamide A (**1**) and stylissoside A (**2**). The potential cytotoxic activities of the isolated compounds are also reported, in addition to the investigation of a possible mechanism of cytotoxic activity through molecular docking simulation studies.

## 2. Results and Discussion

### 2.1. Structure Elucidation of the Isolated Compounds

**Figure 1:**
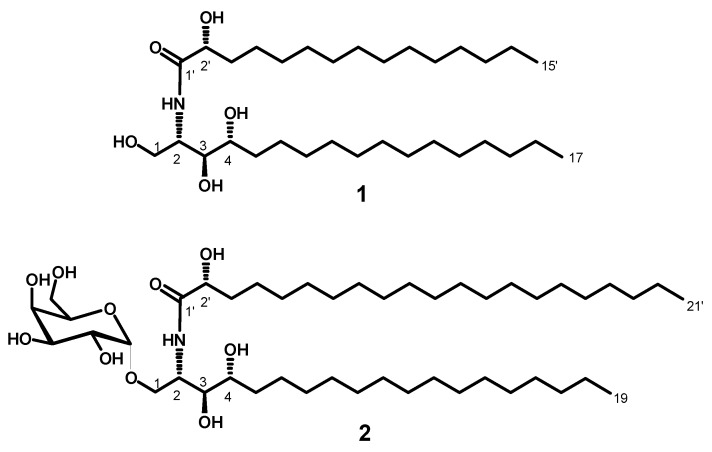
Chemical structures of the newly isolated compounds: stylissamide A (**1**) and stylissoside A (**2**).

Compound **1** (Figure 1) was obtained as a white powder, and its molecular formula was determined to be C_32_H_65_NNaO_5_ by HRESIMS with *m*/*z* 566.4772 [M + Na]^+^ (calcd 566.4760), representing one degree of unsaturation (Supplementary Materials, Figure S1). The ^1^H and ^13^C NMR spectral data of compound **1** are listed in Table 1 (Supplementary Materials, Figures S2–S7). The ^1^H NMR spectrum (measured in C_5_D_5_N, 400 MHz), displayed resonances of an amide proton doublet at *δ*_H_ 8.95 (d, *J* = 8.4 Hz) and a long methylene chain’s protons at *δ*_H_ 1.25, representing a sphingolipid skeleton. Characteristic resonances of the hydrocarbon chain unit 2-amino-1,3,4,2′-tetrol were observed at *δ*_H_ 5.12 (m), (dd, *J* = 8.0, 4.8 Hz), 4.43 (dd, *J* = 8.0, 4.8 Hz), 4.29 (m), 4.62 (m), and 4.37 (m) assigned to H-2, H-1, H-3, H-4, and H-2′, respectively. Resonances corresponding to the aliphatic methyl groups at *δ*_H_ 0.85 (t, *J* = 6.8 Hz) are assigned to CH_3_-17 and CH_3_-15′. 

**Figure 2:**
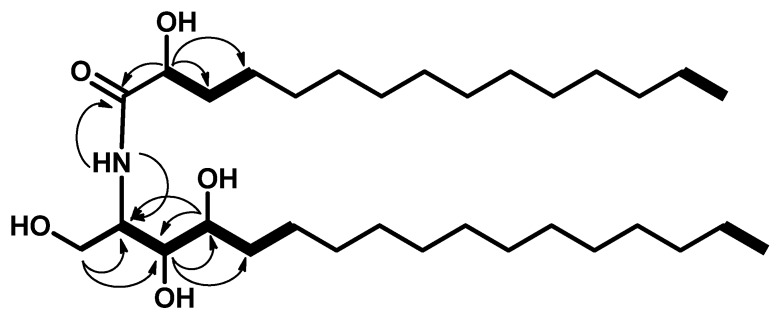
Key ^1^H-^1^H COSY (bold) and HMBC (arrows) correlations in compound **1**.

The ^13^C NMR spectrum (C_5_D_5_N, 100 MHz) of **1** showed 32 carbon signals. Characteristic resonances of a 2-amino-1,3,4,2′-tetrol unit of the hydrocarbon chain were observed at *δ*_C_ 52.7 (C-2), 61.8 (C-1), 76.5 (C-3), 72.7 (C-4), and 72.2 (C-2′). In addition, there was a resonance at *δ*_C_ 14.5 assigned for the two terminal methyl groups (C-17 and C-15′) and at *δ*_C_ 175.0 assigned for the amide carbonyl (C-1′). Analysis of the ^1^H-^1^H COSY, HMQC, and HMBC (Supplementary Materials, Figures S8–S10) spectra led to the assignment of all proton and carbon signals for compound **1**. The positions of the hydroxy groups were confirmed by ^1^H-^1^H COSY correlations between 2H-1/H-2, H-2/H-3, H3/H-4, H-4/H-5, and H-2′/H-3′ and by the HMBC correlations of 2H-1/C-2, 2H-1/C-3, H-3/C-4, H-3/C-5, H-4/C-2, H-4/C-3, H-2/C-1′, and H-2′/C-1′, leading to the assignment of C-1, C-2, C-3, C-4, C-1′, and C-2′ (Figure 2).

The fatty acid length was determined on the basis of the results of its methanolysis followed by peak detection by HRMS, which showed a molecular ion peak at 295.2249 [M + Na]^+^ (calcd for C_16_H_32_NaO_3_, *m*/*z* 295.2249) indicating a C_15_ fatty acid methyl ester for compound **1**. The ceramide moieties’ configuration was assigned by comparing its physical data, ^1^H NMR and ^13^C NMR (measured in C_5_D_5_N) values with those of its analogs, (likewise using pyridine) reported in the literature, wherein the optical rotation +17.4 (*c* 1.00, MeOH) and the chemical shifts of C-2 (*δ* 52.7), C-3 (*δ* 76.5), C-4 (*δ* 72.7), and C-2′ (*δ* 72.2) in addition to the chemical shifts of their corresponding protons were in good agreement with those of phytosphingosine-type ceramides possessing (2*S*, 3*S*, 4*R*, and 2′*R*) configurations [18,19,20]. This evidence suggested the relative configurations of C-2, C-3, C-4, and C-2′ to be 2*S*, 3*S*, 4*R*, and 2′*R*, respectively. Accordingly, the full structure of **1** was assigned as (*R*)-2′-hydroxy-*N*-[(2*S*,3*S*,4*R*)-1,3,4-trihydroxyheptadecan-2-yl]pentadecanamide (stylissamide A), which, to the best of our knowledge, is a new compound.

**Figure 3:**
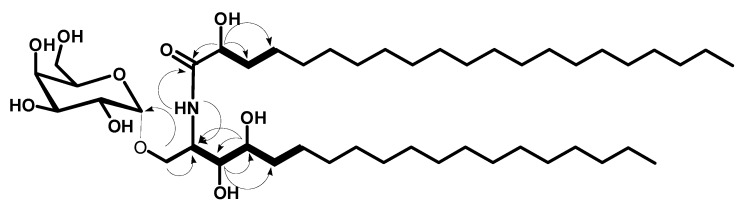
Key ^1^H-^1^H COSY (bold) and HMBC (arrows) correlations for compound **2**.

Compound **2** (Figure 1) was obtained as a white amorphous powder, and its molecular formula was determined to be C_46_H_91_NNaO_10_ by HRESIMS at *m*/*z* 840.6547 [M + Na]^+^ (calcd for *m*/*z* 840.6541), representing one degree of unsaturation (Supplementary Materials, Figure S11). The ^1^H and ^13^C NMR spectral data of compound **2** are listed in Table 1 and in the Supplementary Materials, (Figures S12–S18). The ^1^H NMR spectrum in (C_5_D_5_N, 400 MHz) displayed resonances of an amide proton doublet at *δ*_H_ 8.48 (1H, d, *J* = 8.4 Hz) and a long methylene chain’s protons at *δ*_H_ 1.25, representing a sphingolipid skeleton. Characteristic resonances of a 2-amino-1,3,4,2′-tetrol unit of the hydrocarbon chain were observed at *δ*_H_ 5.29 (1H, m), 4.63 (1H, m), 4.32 (1H, m), 4.59 (1H, m), 4.39 (1H, m), and 4.28 (1H, m) assigned for H-2, H-2′, H-1b, H-1a, H-3, and H-4 respectively. In addition, resonances corresponding to aliphatic methyl groups at *δ*_H_ 0.85 (3H, t, *J* = 6.8 Hz) assigned for CH_3_-19 and CH_3_-21′. The ^13^C NMR spectrum in (C_5_D_5_N, 100 MHz), showed 46 carbon signals. Characteristic resonances of a 2-amino-1,3,4,2′-tetrol unit of the hydrocarbon chain were observed at *δ*_C_ 50.4 (C-2), 72.4 (C-2′), 68.2 (C-1), 76.5 (C-3), and 72.3 (C-4). In addition, there was a resonance at *δ*_C_ 14.2 assigned for the two terminal methyl groups (C-19 and C-21′) and at *δ*_C_ 175.0 attributed to the amide carbonyl (C-1′). The ^13^C NMR spectrum revealed the presence of an anomeric carbon *δ*_C_ 101.2 together with the characteristic signals at *δ*_C_ 70.2, 71.6, 71.0, 73.1, and 62.6 indicating the presence of a sugar moiety. The structure of compound **2** was characterized by comparison of its ^13^C NMR spectral data with those of the known cerebroside, agelasphin, possessing a 2-hydroxy fatty acid group [21,22]. The ^1^H NMR signal at *δ*_H_ 5.61 (d, *J* = 3.4 Hz) clearly indicated that the galactose had an α-linkage [21]. Analysis of the ^1^H-^1^H COSY, HMQC, and HMBC (Supplementary Materials, Figures S19–S21) spectra led to the assignment of the proton and carbon signals for compound **2**. The positions of the hydroxy groups were confirmed by ^1^H-^1^H COSY correlations between 2H-1/ H-2, H-2/H-3, H-3/H-4, H-4/2H-5, and H-2′/2H-3′ and from HMBC correlations of 2H-1/C-2, 2H-1/C-3, H-3/C-4, H-3/C-5, H-4/C-2, H-4/C-3, H-2/C-1′, and H-2′/C-1′, leading to the assignment of C-1/C-2/C-3/C-4/C-1′/C-2′ (Figure 3).

In a similar way as for compound **1**, the chain length of the fatty acid was determined based on the results of its methanolysis followed by detecting peaks from HRMS. The HRMS showed one molecular ion peak at 379.3188 [M + Na]^+^ (calcd for C_22_H_44_NaO_3_: 379.3188) indicating the presence of a C_21_ fatty acid methyl ester for compound **2**. As for compound **1**, The cerebroside’s relative configuration was suggested to be (2*S*, 3*S*, 4*R*, 2*′R*), since the optical rotation +17.40 (*c* 1.00, MeOH), the afore mentioned ^1^H NMR (H-2, H-3, H-4, H-2′) and ^13^C NMR signals (C-1, C-2, C-3, C-4, C-2′) were in good agreement with those of phytosphingosine-type cerebroside molecular species possessing (2*S*,3*S*,4*R*,2*′R*)-configuration [23,24]. So, the full structure of **2** was determined to be (*R*)-*N*-[(2*S*,3*S*,4*R*)-3,4-dihydroxy-1-{[(2*R*,3*R*,4*S*,5*S*,6*R*)-3,4,5-trihydroxy-6-(hydroxymethyl)tetrahydro-2*H*-pyran-2-yl)oxy]nonadecan-2-yl}-2′-hydroxyhenicosanamide (stylissoside A), which, to the best of our knowledge, is a new compound.

### 2.2. Metabolomic Profiling

**Figure 4:**
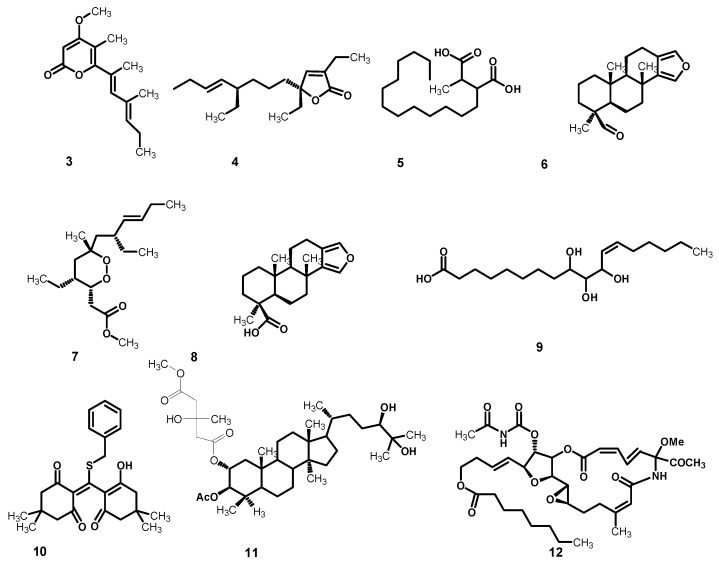
Chemical structures of the annotated metabolites from *Stylissa carteri*, cyercene (**3**), plakortone G (**4**), pedicellic acid (**5**), spongia-13(16),14-dien-19-al (**6**), plakortin (**7**), spongia-13(16),14-dien-19-oic acid (**8**), 9,10,11-trihydroxy-(12*Z*)-12-octadecenoic acid (**9**), benzylthiocrellidone (**10**), methyl aeruginosate C (**11**), and salarin B (**12**).

Metabolomics is a fast growing technology that has effectively contributed to a number of plant-related sciences and drug discovery. The secondary metabolomes of sponges consist of widespread chemically distinct metabolites that represent the outcome of gene expressions in cells, and thus are valuable in recognizing different phenotypic traits [25]. In this context, metabolomic profiling of *Stylissa carteri* using LC–HRESIMS for dereplication purposes (Figure 4) led to the identification of a range of metabolites, mostly represented by oleanane saponins, phenolic diterpenes, and lupane triterpenes. The phytochemicals (Table 2) were tentatively identified by searching in some databases, e.g., the Dictionary of Natural Products (DNP) and METLIN [26,27].

### 2.3. Evaluation of the Antitumor Activity In Vitro

The potential cytotoxicity of compounds **1** and **2** isolated from *Stylissa carteri* was measured by the sulphorhodamine B (SRB) assay adopting the method of Skehan et al. [37] following the protocol described by Vichai and Kirtikara [38] on breast (MCF-7) and liver (HepG2) cancer cell lines. Color intensity was measured on an ELISA reader and the respective IC_50_ (concentration of the compound which reduces survival of cancer cells to 50%) values were calculated. As presented in Table 3, both compounds resulted in promising anticancer activities against breast (MCF-7) and hepatic (HEPG2) cancer cell lines. Compound **1** exhibited stronger cytotoxicity against MCF-7 with an IC_50_ value of 21.1 ± 0.17 µM, while compound **2** displayed a slightly lower cytotoxicity, with an IC_50_ value of 27.5 ± 0.18 µM. The case was reversed in HepG2 cancer cells, where compound **2** was more active (IC_50_ 30.5 ± 0.23 µM) than **1** (IC_50_ 36.8 ± 0.16 µM). The inhibitory properties of these compounds were compared with those of the standard drug cisplatin.

### 2.4. In Silico Studies

**Figure 5:**
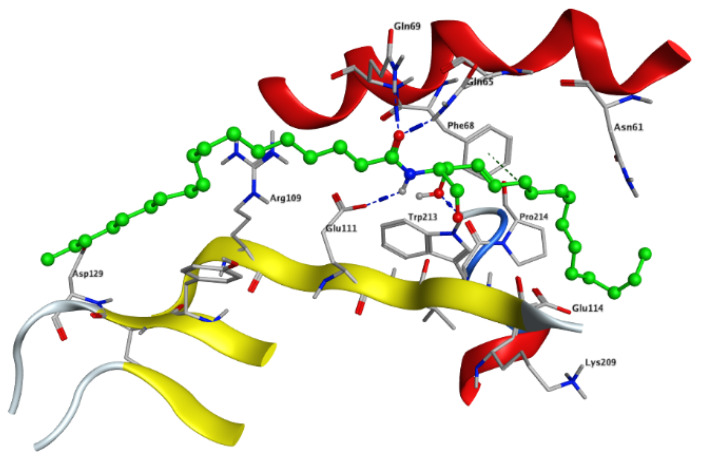
The top generated pose of the induced fit docking simulation oriented C_18_ ceramide (green) in an extended conformation along the space between the helix of the dimerization domain and the β sheets of the SET structure, which was further used as a generated active site for the docking simulations of compounds **1** and **2** within the SET protein.

**Figure 6:**
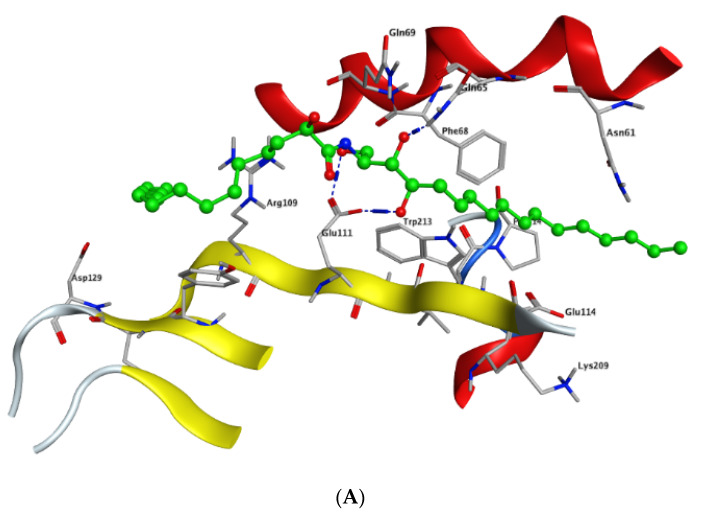

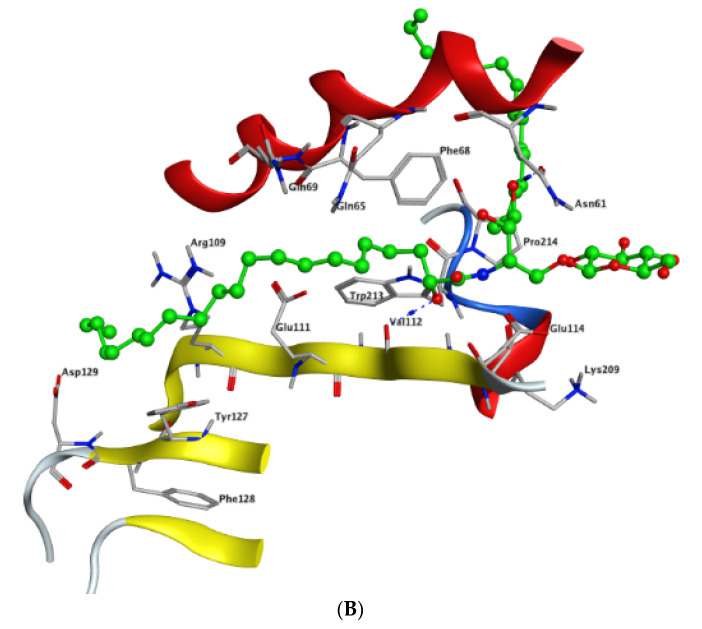
Docking of compounds **1** (**A**) and **2** (**B**) within the SET oncoprotein active site.

Modeling and docking simulations of the newly isolated compounds (**1** and **2**) were performed using the Molecular Operating Environment (MOE) software [39] and the crystal structure of Su(var)3–9, enhancer of Zeste, Trithorax (SET)/inhibitor 2 of protein phosphatase 2A (I2PP2A) oncoprotein; (PDB: 2E50) [40]. D-*erythro*(*e*)-C_18_ ceramide was chosen as a reference compound in the simulation studies due to its higher affinity for binding to SET protein compared to other endogenous ceramides or sphingosine [41]. PP2A has been reported to exhibit a tumor suppressive function by inducing apoptosis or programmed cell death in various cancerous cells [42]. Sphingolipid ceramide has long been described to activate PP2A through a direct interaction with the PP2AC catalytic domain [43], but its mechanistic details have so far remained unknown. Alternatively, another mechanism of ceramide-induced activation of PP2A has been recently found to proceed through the direct binding of ceramide with SET oncoprotein, which functions as an inhibitor of tumor suppressor PP2A [41]. Because the crystal structure of the ceramide-SET complex was not available in the protein data bank, induced fit docking was performed using the crystal structure of the SET protein and C_18_ ceramide as a flexed ligand. The top generated pose (*S* = −4.5237 kcal/mol) of the initial flexible docking revealed that the C_18_ ceramide chain of **1** interacts at the hydrophobic binding pocket along the space between the helix of the dimerization domain and the β sheets within the protein structure. The pocket is predominately lipophilic with some regions of hydrophilicity (Figure 5). Furthermore, our simulation results showed that the ceramide molecule adopts an “extended” conformation inside the SET protein, in which the sphingosine and lipid chains are placed in opposite directions. This finding was further confirmed by the NMR model suggested previously by De Palma et al. [41]. The docking results of compound **1** revealed that the ligand exhibited a similar extended orientation and comparable binding interactions within the pocket (*S* = −6.6821 kcal/mol). The lipid chain of compound **1** forms hydrophobic interactions with the amino acid residues Phe-68, Tyr-127, Pro-214, and Trp-213. Additionally, along the N-terminal region, the 1,4-dihydroxy array of the dihydrosphingosine chain donates two H-bonds to Glu-111 (2.843 and 2.849 Å, respectively), while the 3-hydroxy group accepts an H-bond from Gln-65 with 2.138 Å (Figure 6A). Previous data had indicated that the amino acid residues 36–124 of the N-terminal region are involved in the inhibition of PP2A as well as the binding with ceramide [41]. On the other hand, compound **2** was found to exhibit a different compact conformation within the active site, where the dihydrosphingosine backbone runs perpendicular to both the fatty acyl chain and the sugar moiety. Therefore, compound **2** showed a weaker binding pattern within the pocket (*S* = −9.2672 kcal/mol) (Figure 6B). The lipid chain of compound **2** forms hydrophobic interactions with the amino acid residues Phe-68, Phe-67, and Pro-214. The longer chains of compound **2** destabilize the complex by shifting the ligand from the main binding sites within the SET structure. The hydroxy group of dihydrosphingosine chain donates an H-bond to the amide carbonyl group of Val-112 (2.78 Å), the 4-OH of lipid chain donates an H-bond to Asn-61 (2.77 Å), and the CH_2_OH of the sugar moiety projects out the active site and donates an H-bond to Glu-116 (2.91 Å).

## 3. Materials and Methods

### 3.1. General Experimental Procedures

^1^H NMR (400 MHz), ^13^C NMR (100 MHz), DEPT-135 and 2D NMR spectra were registered on a Varian AS 400 (Varian Inc., Palo Alto, CA, USA) using the residual solvent signal as an internal standard. High-resolution mass spectra were recorded using a Bruker BioApex (Bruker Corporation) machine. Pre-coated silica gel G-25 UV254 plates were used for thin layer chromatography (TLC) (20 cm × 20 cm) (E. Merck, Darmstadt, Germany). Silica gel (Purasil 60A, 230–400 mesh) was used for flash column chromatography (Whatman, Sanford, ME, USA).

### 3.2. Sponge Material

The sponge *Stylissa carteri* was collected from Sharm El Sheikh in the Egyptian Red Sea. The sponge material was air-dried and kept at low temperature (−24 °C) before further processing. Identification of the sponge was performed by Dr. Tarek Temraz, Marine Science Department, Faculty of Science, Suez Canal University, Ismailia, Egypt. Voucher specimens were deposited under registration number SAA-46 in the herbarium section of Pharmacognosy Department, Faculty of Pharmacy, Suez Canal University, Ismailia, Egypt.

### 3.3. Extraction and Isolation

*Stylissa carteri* sponge (1.5 kg) was frozen, chopped to small pieces, and then extracted with methanol (3 × 2 L) at room temperature. The combined extract was concentrated in vacuo to give a reddish-brown viscous residue (30 g), which was suspended in distilled water (1 L) and partitioned with *n*-hexane, chloroform, and *n*-butanol giving rise to four fractions, namely SC-1 (4 g), SC-2 (3 g), SC-3 (17.8 g), and SC-4 (3.5 g). Fractions SC-1 and SC-2 were concentrated and combined to give SC-C (7 g), which was chromatographed on a silica gel column using CHCl_3_: MeOH (1:0 ~ 6.5:3.5) affording eight sub-fractions (SC-C-1 - SC-C-8). Subfraction SC-C-2 (2.6 g) was chromatographed on a silica gel column using CHCl_3_: MeOH (1:0 ~ 6.5:3.5) followed by a two-step purification on Sephadex LH-20 under isocratic conditions (CHCl_3_:MeOH 1:1) to obtain pure compound **1** (25 mg, white amorphous powder). Subfraction SC-C-6 (450 mg) was chromatographed on a silica gel column using an isocratic elution of CHCl_3_:MeOH (9:1 ~ 6.5:3.5) followed by a Sephadex LH-20 column using isocratic elution conditions of CHCl_3_:MeOH (1:1) to get compound **2** (31 mg, white amorphous powder) in a pure form.

(*R*)-2′-Hydroxy-*N*-[(2*S*,3*S*,4*R*)-1,3,4-trihydroxyheptadecan-2-yl]pentadecanamide, stylissamide A (**1**): white, amorphous powder; ^1^H NMR and ^13^C NMR (see Table 1); HRESIMS (positive ion mode) *m*/*z* 566.4772 [M + Na]^+^ (calcd for C_32_H_65_NNaO_5_, 566.4760). 

(*R*)-*N*-{(2*S*,3*S*,4*R*)-3,4-Dihydroxy-1-[(2*R*,3*R*,4*S*,5*S*,6*R*)-3,4,5-trihydroxy-6-(hydroxymethyl)tetrahydro-2H-pyran-2-yl)oxy]nonadecan-2-yl}-2′-hydroxyhenicosanamide, stylissoside A (**2**): white, amorphous powder; ^1^H NMR and ^13^C NMR (see Table 1); HRESIMS (positive ion mode) *m*/*z* 840.6547 [M + H]^+^ (calcd for C_46_H_91_NNaO_10_, 840.6541).

### 3.4. Ceramide Hydrolysis

An aliquot of 5 mg of **1** and **2** were heated with 5% HCl/MeOH (0.5 mL) at 70 °C for 8 h. The mixture was extracted with *n*-hexane and concentrated in vacuo to afford the corresponding hydroxy fatty acid methyl esters. The HRMS of **1** showed a molecular ion peak at *m*/*z* 295.2249 [M + Na]^+^ (calcd for C_16_H_32_NaO_3_: 295.2249) indicating the presence of a C_15_ fatty acid methyl ester. For compound **2**, the HRMS analysis showed a molecular ion peak at *m*/*z* 379.3188 [M + Na]^+^ (calcd for C_22_H_44_NaO_3_: 379.3188) evidencing a C_21_ fatty acid methyl ester.

### 3.5. Identification of the Sugar Moiety in Compound ***2***

Compound **2** (5 mg) was heated at 70 °C with 5% HCl/MeOH (0.5 mL) for 8 h then the reaction mixture (including the esterified fatty acid) was extracted with chloroform. The methanolic layer (containing the sugar moiety) was neutralized with Ag_2_CO_3_. The resulting precipitates were filtered off and the filtrate was concentrated in vacuo then injected on HPLC (Cosmosil Sugar-D, 4.6 ID × 250 mm, 1 mL/min, RI detector, 95% aqueous acetonitrile). HPLC runs of standard glucose and galactose were carried out in parallel and the respective retention times were compared with that of the unknown sugar in **2**. Galactose showed a retention time of 4.76 min, similar to that of the sugar in compound **2**, however, glucose displayed a retention time of 4.62 min, which was eluted earlier from the sugar in **2**. Therefore, the sugar in the new compound **2** was identified as galactose.

### 3.6. Determination of the Configuration of the Sugar Moiety in ***2***

Compound **2** (2 mg) was hydrolyzed by heating in 0.5 M aqueous HCl (0.1 mL) and then neutralized with Amberlite IRA400. After drying in vacuo, the residue was dissolved in pyridine (0.1 mL) containing L-cysteine methyl ester hydrochloride (0.5 mg) and heated at 60 °C for 1 h in 0.1 mL solution of *o*-tolyl isothiocyanate (0.5 mg) in pyridine. The reaction mixture was directly analyzed by reversed-phase HPLC (Cosmosil-5-C_18_-AR-II, 4.6 ID × 250 mm, 0.8 mL/min, λ = 250 nm, 25% acetonitrile in 50 mM H_3_PO_4_). The peaks at 19.58 min and 17.52 min coincided with those of the respective derivatives of D-galactose. The same derivatization procedures were performed on 5 mg of the standard compounds, D-galactose (*t*_R_ = 18.6 min) and L-galactose (*t*_R_ = 19.3 min).

### 3.7. Metabolomic Profiling

The metabolomics profiling was performed according to a method reported by Elsayed et al. [44]. These files were imported to the data mining software MZmine 2.10 for peak picking, deconvolution, deisotoping, alignment, and formula prediction. Dereplication of compounds was carried out by comparison with those in the Marinlit database and the Dictionary of Natural Products (DNP) 2015.

### 3.8. Cytotoxicity Assays

The cytotoxicity of compounds **1** and **2** was measured by the sulphorhodamine B (SRB) assay as described by Skehan [37], following the protocol described by Vichai and Kirtikara [38] on breast (MCF-7) and liver (HepG2) cancer cell lines. At an initial concentration of 3 × 10^3^ cell/well in a 150 μL fresh medium, cells were seeded in 96-well microtiter plates, and left for 24 h to adhere to the plates. Different concentrations of the drug were added 0, 5, 12.5, 25, and 50 μg/mL respectively.

The plates were incubated for 48 h. The cells were fixed at 4 °C with 50 μL cold trichloroacetic acid (10% final concentration) for 1 h. The plates were washed with distilled water (automatic washer Tecan, Germany) and stained with 50 μL 0.4% SRB dissolved in 1% acetic acid at room temperature for 30 minutes. With 1% acetic acid, the plates were washed and air-dried. 100 μL/well of 10 M tris base (pH 10.5) was used to solubilize the dye. Using an ELISA microplate reader (Sunrise Tecan reader, Germany), the optical density of each well was measured at 570 nm spectrophotometrically. The mean background absorbance was automatically subtracted, and mean values were determined for each concentration of drugs. The experiment was repeated three times, then the IC_50_ values were calculated.

### 3.9. In Silico Studies

All the molecular modeling calculations and docking simulation studies were performed using the Molecular Operating Environment (MOE 2014.0901, 2014; Chemical Computing Group, Canada) software. All MOE minimizations were performed until an RMSD gradient of 0.01 kcal/mol/Å with the force field (Amber10:ETH) and gas phase solvation to calculate the partial charges automatically. Before simulations, the protein was protonated using the LigX function and the monomer was identified. Induced fit docking simulation was performed initially to predict the active site using C_18_ ceramide as a flexed ligand. Triangle matching with London dG scoring was chosen for initial placement, then the top 30 poses were refined using force field (Amber10:ETH) and Affinity DG scoring. The top pose from this simulation was analyzed and further used as a reference for the docking simulation of the newly isolated compounds **1** and **2**. The output database dock file was created with different poses for each ligand and arranged according to the final score function (S), which is the score of the last stage that was not set to zero.

## 4. Conclusions

The present research highlighted the efficacy of LCMS profiling when combined with bioassay-guided drug discovery from marine invertebrates to accelerate the conventionally long processes of identifying an active metabolite by successive isolation from crude extracts. Dereplication experiments focused on the chemotaxonomic sorting helped in the identification of putative active metabolites, while structural assignment of the isolated compounds, using both HRMS and NMR, supported the identified hits. Metabolomic profiling of the bioactive extract displayed the existence of numerous secondary metabolites, mainly oleanane saponins and phenolic and lupane di- and triterpenes. Therefore, bio-guided isolation combined with LC-MS metabolomic profiling of the Red Sea sponge *Stylissa carteri* crude extract was performed, leading to the characterization of two new compounds, stylissamide A (**1**) and stylissoside A (**2**), which, to the best of our knowledge, have not been isolated from any natural source before. Both compounds, showed promising cytotoxic activity against breast (MCF-7) and liver (HepG2) cancer cell lines. Compound **1** exhibited stronger cytotoxicity against breast (MCF-7) cancer cells, while compound **2** exhibited higher activity against the HepG2 cancer cell line compared to cisplatin as the standard. Moreover, a docking study was performed to investigate the possible mechanism(s) of the cytotoxic activity of **1** and **2**. The newly isolated metabolites were docked into the crystal structure of the SET oncoprotein and inhibitor 2 of protein phosphatase 2A (I2PP2A). We believe that the in vivo biological investigations of these metabolites will be of value for future anti-cancer drug development.

## Figures and Tables

**Table 1:** ^1^H (400 MHz) and ^13^C NMR (100 MHz) for the new compounds **1** and **2** in C_5_D_5_N.

1			2		
Position	*δ_H_* (mult., *J*_Hz_)	*δ_C_*	Position	*δ_H_* (mult., *J*_Hz_)	*δ_C_*
1	4.43, dd (8.0, 4.8)	61.8	1a	4.32, m	68.2
2	5.12, dd (8.0, 4.8)	52.7	1b	4.59, m
3	4.29, m	76.5	2	5.29, m	50.4
4	4.62, m	72.7	3	4.39, m	76.5
5	1.25, m	30.2	4	4.28, m	72.3
6	1.25, m	30.0	5	1.27, m	31.9
7–14	1.25, m	29.7	6	1.27, m	30.2
15	1.25, m	29.9	7-18	1.27, m	29.9
16	1.25, m	22.7	19	0.85, t (6.8)	14.2
17	0.85, t (6.8)	14.1	1′	-	175.0
1′	-	175.0	2′	4.63, m	72.4
2′	4.37, m	72.2	3′	2.00, m	31.9
3′	2.05, m	30.2	4′	1.27, m	30.2
4′	1.25, m	30.0	5′–20′	1.27, m	29.9
5′13′	1.25, m	29.7	21′	0.85, t (6.8)	14.2
14′	1.25, m	22.7	NH	8.53, d (8.4)	-
15′	0.85, t (6.8)	14.1	1′′	5.61, d (3.4)	101.2
NH	8.59, d (8.4)		2′′	4.64, m	70.2
			3′′	4.50, m	71.6
			4′′	4.54, m	71.0
			5′′	4.50, m	73.1
			6′′	4.33, m	62.6

**Table 2:** Dereplicated metabolites from *Stylissa carteri*.

	RT (min)	MZMine ID	Molecular Weight	Name	Source	Reference
1	5.108521	209	234.1261	Cyercene (**3**)	Mollusca *Cyerce cristallina*	[28]
2	10.04907	57	278.2253	Plakortone G (**4**)	Porifera *Plakortis sp*	[29]
3	8.3637	13	314.2445	Pedicellic acid (**5**)	*Didymocarpus pedicellate*	[30]
4	10.10114	263	300.2095	Spongia-13(16),14-dien-19-al (**6**)	Porifera *Spongia officinalis*	[31]
5	7.916396	12	312.2289	Plakortin (**7**)	*Plakortis halichondrioides, Sponge*	[32]
6	8.382825	227	316.2043	Spongia-13(16),14-dien-19-oic acid (**8**)	Porifera *Spongia officinalis*	[31]
7	6.245154	20	330.2394	9,10,11-Trihydroxy-(12*Z*)-12-octadecenoic acid (**9**)	Chinese truffle *Tuber indicum*	[33]
8	3.509688	291	412.1711	Benzylthiocrellidone (**10**)	Porifera *Crella spinulata*	[34]
9	13.7787	225	676.4528	Methyl aeruginosate C (**11**)	*Stropharia aeruginosa*	[35]
10	13.4309	228	720.3484	Salarin B (**12**)	Porifera *Fascaplysinopsis* sp	[36]

**Table 3:** IC_50_ values of compounds 1 and 2 on breast (MCF-7) and liver (HEPG2) cancer cell lines.

	HepG2	MCF-7
	IC_50_ (µM)	
**1**	36.8 ± 0.16	21.1 ± 0.17
**2**	30.5 ± 0.23	27.5 ± 0.18
Cisplatin	21.3 ± 0.40	15.3 ± 0.10

Each data point represents the mean ± SD of four independent experiments (significant differences at *p* < 0.05).

## Supplementary Materials

The following are available online at https://www.mdpi.com/1660-3397/18/5/241/s1, Figures S1–S10: HRMS, ^1^H NMR, ^13^C NMR, and COSY, HMBC, and HMQC of Compound **1**, Figures S11–S21: HRMS, ^1^H NMR, ^13^C NMR, and COSY, HMBC, and HMQC of Compound **2**.

## References

[1] Fu Y., Luo J., Qin J., Yang M. (2019). Screening techniques for the identification of bioactive compounds in natural products. J. Pharm. Biomed. Anal..

[2] Abdelhameed R.F., Ibrahim A.K., Temraz T.A., Yamada K., Ahmed S.A. (2017). Chemical and biological investigation of the Red Sea sponge Echinoclathria species. Int. J. Pharm. Sci. Res..

[3] Liu M., El-Hossary E.M., Oelschlaeger T.A., Donia M.S., Quinn R.J., Abdelmohsen U.R. (2019). Potential of marine natural products against drug-resistant bacterial infections. Lancet Infect. Dis..

[4] Hifnawy M.S., Aboseada M.A., Hassan H.M., Tohamy A.F., Abdel-Kawi S.H., Rateb M.E., El Naggar E.B., Quinn R.J., Abdelmohsen U.R. (2020). Testicular caspase-3 and β-Catenin regulators predicted via comparative metabolomics and docking studies. Metabolites.

[5] Abdelmohsen U.R., Balasubramanian S., Oelschlaeger T.A., Grkovic T., Pham N.B., Quinn R.J., Hentschel U. (2017). Potential of marine natural products against drug-resistant pathogens. Lancet Infect. Dis..

[6] Khalifa S.A.M., Elias N., Farag M.A., Chen L., Saeed A., Hegazy M.E.F., Moustafa M.S., Abd El-Wahed A., Al-Mousawi S.M., Musharraf S.G. (2019). Marine natural products: A source of novel anticancer drugs. Mar. Drugs.

[7] Anjum K., Abbas S.Q., Shah S.A., Akhter N., Batool S., Shams ul-Hassan S. (2016). Marine sponges as a drug treasure. Biomol Ther..

[8] Sayed A.M., Alhadrami H.A., El-Hawary S.S., Mohammed R., Hassan H.M., Rateb M., Abdelmohsen U.R., Bakeer W. (2020). Discovery of two brominated oxindole alkaloids as Staphylococcal DNA gyrase and pyruvate kinase inhibitors via inverse virtual screening. Microorganisms.

[9] El-Hawary S.S., Sayed A.M., Mohammed M., Hassan H.M., Rateb M.E., Amin E., Mohammed T.A., El-Mesery M., Abdullatif Bin Muhsinah A., Alsayari A. (2019). Bioactive brominated oxindole alkaloids from the Red Sea sponge Callyspongia siphonella. Mar. Drugs.

[10] Eltahawy N.A., Ibrahim A.K., Radwan M.M., Zayton S., Gomaa M., ElSohly M.A., Hassanean H.A., Ahmed S.A. (2015). Mechanism of action of antiepileptic ceramide from Red Sea soft coral Sarcophyton auritum. Bioorg. Med. Chem. Lett..

[11] Hannun Y.A., Obeid L.M. (2008). Principles of bioactive lipid signaling: Lessons from sphingolipids. Nat. Rev. Mol. Cell Biol..

[12] Eltahawy N.A., Ibrahim A.K., Gomaa M.S., Zaitone S.A., Radwan M.M., Hassanean H.A., ElSohly M.A., Ahmed S.A. (2019). Anxiolytic and anticonvulsant activity followed by molecular docking study of ceramides from the Red Sea sponge Negombata sp.. Med. Chem. Res..

[13] Giles E.C., Saenz-Agudelo P., Berumen M.L., Ravasi T. (2013). Novel polymorphic microsatellite markers developed for a common reef sponge *Stylissa carteri*. Mar. Biodiv..

[14] Linington R.G., Williams D.E., Tahir A., Van Soest R., Andersen R.J. (2003). Latonduines A and B, new alkaloids isolated from the marine sponge *Stylissa carteri*: Structure elucidation, synthesis, and biogenetic implications. Org. Lett..

[15] Patel K., Laville R., Martin M., Tilvi S., Moriou C., Gallard J., Ermolenko L., Debitus C., Al-Mourabit A. (2010). Unprecedented stylissazoles A–C from *Stylissa carteri*: Another dimension for marine pyrrole-2-aminoimidazole metabolite diversity. Angew. Chem. Int. Ed..

[16] O’Rourke A., Kremb S., Bader T., Helfer M., Schmitt-Kopplin P., Gerwick W., Brack-Werner R., Voolstra C. (2016). Alkaloids from the sponge *Stylissa carteri* present prospective scaffolds for the inhibition of Human Immunodeficiency Virus 1 (HIV-1). Mar. Drugs.

[17] Inbaneson S.J., Ravikumar S. (2012). In vitro antiplasmodial activity of marine sponge *Stylissa carteri* associated bacteria against Plasmodium falciparum. Asian Pac. J. Trop. Dis..

[18] Sun Y., Xu Y., Liu K., Hua H., Zhu H., Pei Y. (2006). Gracilarioside and gracilamides from the red alga Gracilaria asiatica. J. Nat. Prod..

[19] Azuma H., Takao R., Niiro H., Shikata K., Tamagaki S., Tachibana T., Ogino K. (2003). Total syntheses of symbioramide derivatives from L-Serine and their antileukemic activities. J. Org. Chem..

[20] Sandjo L., Hannewald P., Yemloul M., Kirsch G., Ngadjui B. (2008). Triumfettamide and Triumfettoside Ic, two ceramides and other secondary metabolites from the stems of wild Triumfetta cordifolia A. Rich. (Tiliaceae). Helv. Chim. Acta..

[21] Natori T., Morita M., Akimoto K., Koezuka Y. (1994). Agelasphins, novel antitumor and immunostimulatory cerebrosides from the marine sponge Agelas mauritianus. Tetrahedron Lett..

[22] Natori T., Koczuka Y., Higa T. (1993). Agelasphins, novel alpha-galactosylceramides from the marine sponge Agelas mauritianus. Tetrahedron Lett..

[23] Kawatake S., Nakamura K., Inagaki M., Higushi R. (2002). Isolation and structural determination of six glucocerebrosides from the starfish Luidia maculata. Chem. Pharm. Bull..

[24] Chen X., Wu Y., Chen D. (2002). Structure determination and synthesis of a new cerebroside isolated from the traditional Chinese medicine Typhonium giganteum. Engl. Tetrahedron Lett..

[25] Abdelhafez O.H., Othman E.M., Fahim J.R., Desoukey S.Y., Pimentel-Elardo S.M., Nodwell J.R., Schirmeister T., Tawfike A., Abdelmohsen U.R. (2019). Metabolomics analysis and biological investigation of three Malvaceae plants. Phytochem. Anal..

[26] Dictionary of Natural Products. http://dnp.chemnetbase.com/faces/chemical/ChemicalSearch.xhtml.

[27] METLIN. http://metlin.scripps.edu/index.php.

[28] Vardaro R.R., Matzo V.D., Crispino A., Cimino G. (1991). Cyercenes, novel polypropionate pyrones from the autotomizing Mediterranean mollusc Cyerce cristallina. Tetrahedron Lett..

[29] Gochfeld D.J., Hamann M.T. (2001). Isolation and biological evaluation of filiformin, plakortide F, and plakortone G from the Caribbean sponge Plakortis sp.. J. Nat. Prod..

[30] Rao K.V., Seshadri T.R., Sood M.S. (1966). Isolation and constitution of pedicellic acid: A new dicarboxylic acid from the leaves of Didymocarpus pedicellata. Tetrahedron Lett..

[31] Li C., Schmitz F.J., Kelly-Borges M. (1999). Six new spongian diterpenes from the sponge Spongia matamata. J. Nat. Prod..

[32] Martin D., Higgs D., Faulkner J. (1978). Plakortin, an antibiotic from Plakortis halichondrioides. J. Org. Chem..

[33] Gao J., Wang C., Zhang A., Liu J. (2001). A new trihydroxy fatty acid from the ascomycete, Chinese truffle Tuber indicum. Lipids.

[34] Pattenden G., Wickramasinghe W.A., Bandaranayake W.M. (2002). Benzylthiocrellidone, a novel thioether with strong UV A and B absorption from the Great Barrier Reef sponge Crella spinulata (Poecilosclerida: Crellidae). Article.

[35] Shiono Y., Sugasawa H., Kurihara N., Nazarova M., Murayama T., Takahashi K., Ikeda M. (2005). Three lanostane triterpenoids from the fruiting bodies of Stropharia aeruginosa. J. Asian Nat. Prod. Res..

[36] Aknin M., Gros E., Vacelet J., Kashman Y., Gauvin-Bialecki A. (2010). Sterols from the Madagascar sponge Fascaplysinopsis sp.. Mar. Drugs.

[37] Skehan P., Storeng R., Scudiero D., Monks A., McMahn J.M., Vistica D., Warren J., Bokesch H., Kenney S., Boyd M.R. (1990). New colorimetric cytotoxicity assay for anticancer-drug screening. J. Nat. Cancer Inst..

[38] Vichai V., Kirtikara K. (2006). Sulforhodamine B colorimetric assay for cytotoxicity screening. Nat. Protoc..

[39] Chemical Computing Group Inc. (2016). Molecular Operating Environment (MOE) 2014.0901.

[40] Muto S., Senda M., Akai Y., Sato L., Suzuki T., Nagai R., Senda T., Horikoshi M. (2007). Relationship between the structure of SET/TAF-Iβ/INHAT and its histone chaperone activity. Proc. Natl. Acad. Sci. USA.

[41] De Palma R.M., Parnham S.R., Li Y., Oaks J.J., Peterson Y.K., Szulc Z.M., Roth B.M., Xing Y., Ogretmen B. (2019). The NMR-based characterization of the FTY720-SET complex reveals an alternative mechanism for the attenuation of the inhibitory SET-PP2A interaction. FASEB J..

[42] Liu J., Beckman B.S., Foroozesh M. (2013). A review of ceramide analogs as potential anticancer agents. Future Med. Chem..

[43] Mullen T.D., Obeid L.M. (2012). Ceramide and apoptosis: Exploring the enigmatic connections between sphingolipid metabolism and programmed cell death. Anticancer. Agents Med. Chem..

[44] Elsayed Y., Refaat J., Abdelmohsen U.R., Othman E.M., Stopper H., Fouad M.A. (2018). Metabolomic profiling and biological investigation of the marine sponge-derived bacterium Rhodococcus sp. UA13. Phytochem. Anal..

