# Supplementary material for: New Cytotoxic Natural Products from the Red Sea Sponge Stylissa carteri
Source: Mar Drugs. 2020 May 3;18(5):241. doi: 10.3390/md18050241 (PMC7281077; doi:10.3390/md18050241)
Supplement: Supplementary file 1 [file marinedrugs-18-00241-s001.pdf]

Supporting Information

New Cytotoxic Natural Products from the Red Sea Sponge *Stylissa Carteri*

Reda F. A. Abdelhameed [1,†], Eman S. Habib [1, †], Nermeen A. Eltahawy [1], Hashim A. Hassanean [1], Amany K. Ibrahim [1], Anber F. Mohammed [2], Shaimaa Fayez [3,4], Alaa M. Hayallah [2,5], Koji Yamada [6], Fathy A. Behery [7,8], Mohammad M. Al-Sanea [9], Sami I. Alzarea [10], Gerhard Bringmann [3], Safwat A. Ahmed [1,*] and Usama Ramadan Abdelmohsen [11,12,*]

[1] Department of Pharmacognosy, Faculty of Pharmacy, Suez Canal University, 41522 Ismailia, Egypt; omarreda_70@yahoo.com (R.F.A.A.); emy_197@hotmail.com (E.S.H.); nermeenazmy25@gmail.com (N.A.E.); hasanean2000@yahoo.com (H.A.A.); am_kamal66@yahoo.com (A.K.I.)

[2] Department of Pharmaceutical Organic Chemistry, Faculty of Pharmacy, Assiut University, 71526 Assiut, Egypt; anber_pharm_2006@yahoo.com (A.F.M.); alaa_hayalah@yahoo.com (A.M.H.)

[3] Institute of Organic Chemistry, University of Würzburg, Am Hubland, 97074 Würzburg, Germany; shaimaa.seaf@uni-wuerzburg.de (S.F.); bringmann@chemie.uni-wuerzburg.de (G.B.)

[4] Department of Pharmacognosy, Faculty of Pharmacy, Ain-Shams University, 11566 Cairo, Egypt;

[5] Department of Pharmaceutical Chemistry, Faculty of Pharmacy, Deraya University, 61111 New Minia, Egypt;

[6] Graduate School of Biomedical Sciences, Nagasaki University, Bunkyo-machi 1-14, 852-8521 Nagasaki, Japan; kyamada@nagasaki-u.ac.jp

[7] Department of Pharmacognosy, Faculty of Pharmacy, Mansoura University, Mansoura 35516, Egypt; fathybehery@yahoo.com

[8] Department of Pharmaceutical Sciences, College of Pharmacy, Riyadh Elm University, 11681 Riyadh, Saudi Arabia.

[9] Department of Pharmaceutical Chemistry, College of Pharmacy, Jouf University, 72341 Aljouf, Saudi Arabia; mohmah80@gmail.com

[10] Department of Pharmacology, College of Pharmacy, Jouf University, 72341 Aljouf, Saudi Arabia; samisz@ju.edu.sa

[11] Department of Pharmacognosy, Faculty of Pharmacy, Deraya University, 61111 New Minia, Egypt,

[12] Department of Pharmacognosy, Faculty of Pharmacy, Minia University, 61519 Minia, Egypt,

† Equal contributions: Eman S. Habib and Reda F.A. Abdelhameed as first author.

* Correspondence: usama.ramadan@mu.edu.eg (U.R.A.) and safwat_aa@yahoo.com (S.A.A)

Table of Contents

Figure S1: HRMS of Compound **1** (+MS**)**…………………………………………………………..………………..……..……....3
Figure S2: ^{1}H-NMR spectrum of compound **1** in (C_5D_5N, 400 MHz)…………………………………………………………..4
Figure S3: Partial expansions of the ^{1}H-NMR spectrum of compound **1** in (C_5D_5N, 400 MHz)…………………………………..5
Figure S4: Partial expansions of the ^{1}H-NMR spectrum of compound **1** in (C_5D_5N, 400 MHz) **…**…………………………..……........6
Figure S5: ^{13}C-NMR spectrum of compound **1** in (C_5D_5N, 100 MHz).…………………..………………….……………....7
Figure S6: Partial expansion of the ^{13}C-NMR spectrum of compound **1** in (C_5D_5N, 100 MHz)…..8
Figure S7: Partial expansion of the ^{13}C-NMR spectrum of compound **1** in (C_5D_5N, 100 MHz)………..……...................................9
Figure S8: 2D-COSY spectrum of compound **1** in (C_5D_5N, 400 MHz)….……………………………………………..………..10
Figure S9: 2D-HMQC spectrum of compound **1** in (C_5D_5N, 400 MHz)…..……………………….…………………………….…..11
Figure S10: 2D-HMBC spectrum of compound **1** in (C_5D_5N, 400 MHz)….……………………….………………………….…12
Figure S11: HRMS of Compound **2** (+MS**)**…..………………………………………………..………………..………...13
Figure S12: ^{1}H-NMR spectrum of compound **2** in (C_5D_5N, 400 MHz)…………………………………………………………..14
Figure S13: Partial expansions of the ^{1}H-NMR spectrum of compound **2** in (C_5D_5N, 400 MHz)……………………………………..15
Figure S14: Partial expansions of the ^{1}H-NMR spectrum of compound **2** in (C_5D_5N, 400 MHz) **…**…………………………..……........16
Figure S15: ^{13}C-NMR spectrum of compound **2** in (C_5D_5N, 100 MHz).…………………..………………….……………....17
Figure S16: Partial expansion of the ^{13}C-NMR spectrum of compound **2** in (C_5D_5N, 100 MHz)…..18
Figure S17: Partial expansion of the ^{13}C-NMR spectrum of compound **2** in (C_5D_5N, 100 MHz)………..……...................................19
Figure S18: Partial expansion of the ^{13}C-NMR spectrum of compound **2** in (C_5D_5N, 100 MHz)………..……....................................20
Figure S19: 2D-COSY spectrum of compound **2** in (C_5D_5N, 400 MHz)….……………………………………………..………..21
Figure S20: 2D-HMQC spectrum of compound **2** in (C_5D_5N, 400 MHz)…..……………………….…………………………….…..22
Figure S21: 2D-HMBC spectrum of compound **2** in (C_5D_5N, 400 MHz)….……………………….………………………….…23

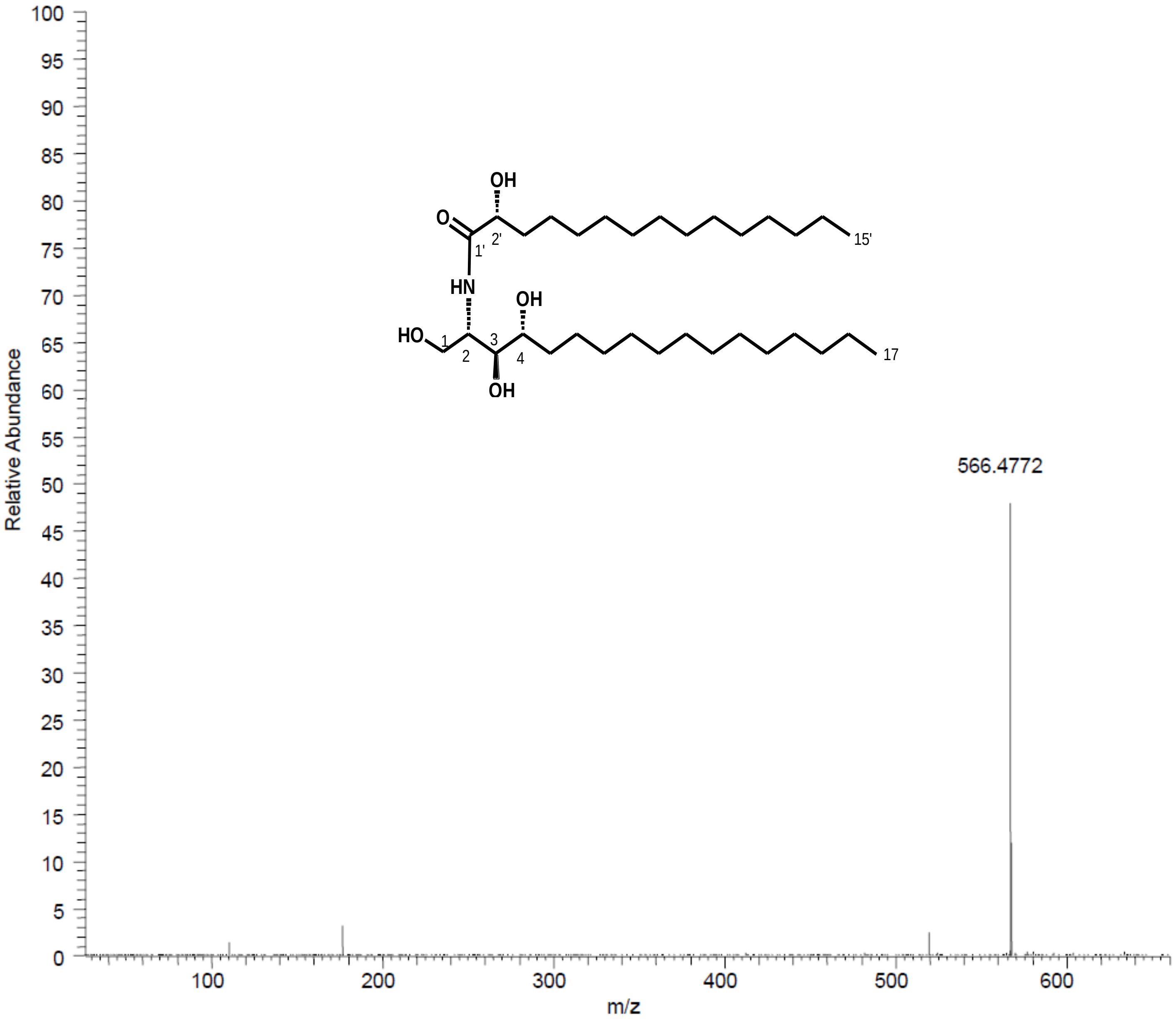

Figure S1: HRMS of Compound **1** (+MS)

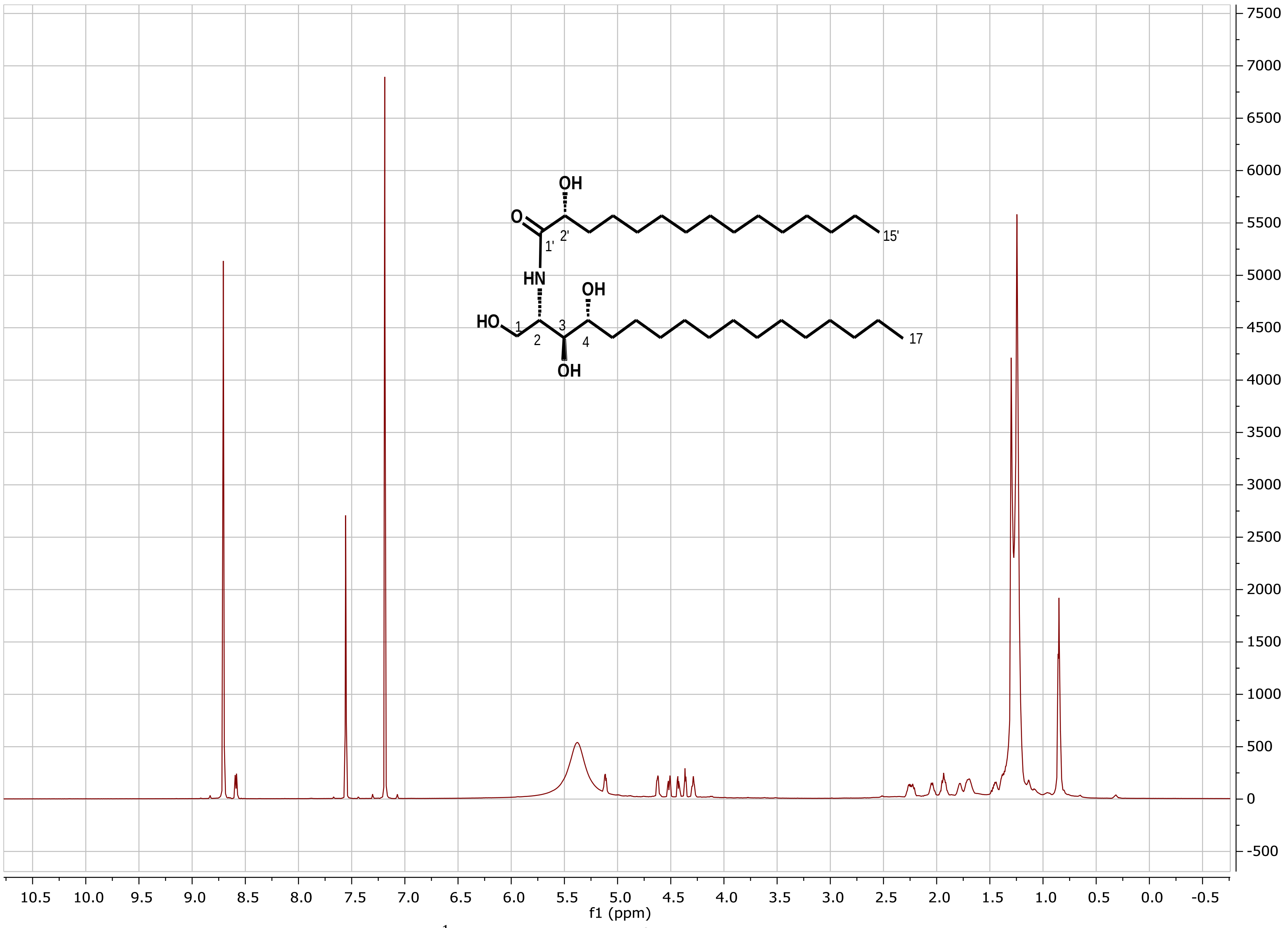

Figure S2: ^{1}H-NMR spectrum of compound **1** in (C_5D_5N, 400 MHz)

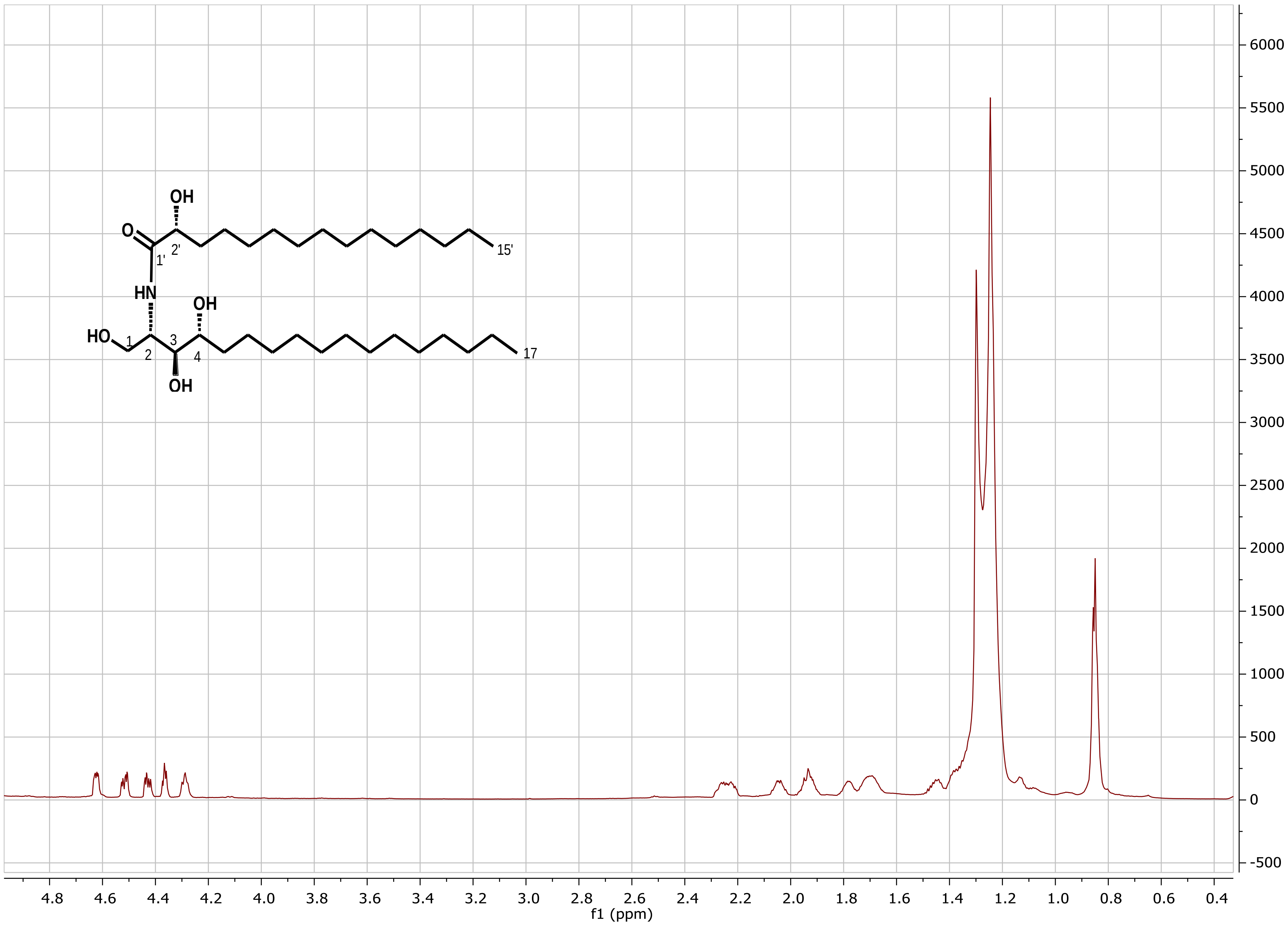

Figure S3: Partial expansions of the ^{1}H-NMR spectrum of compound **1** in (C_5D_5N, 400 MHz)

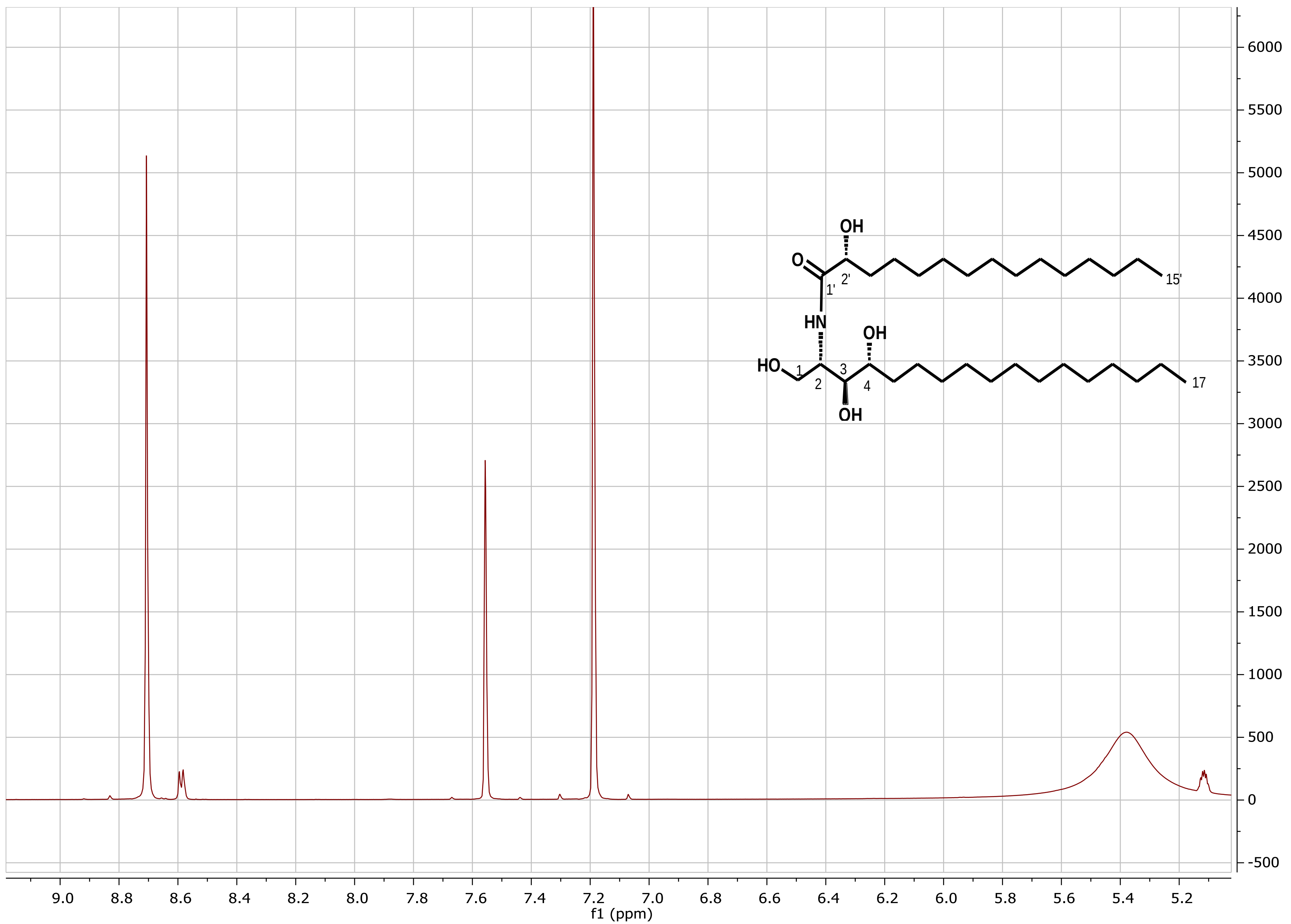

Figure S4: Partial expansions of the ^{1}H-NMR spectrum of compound **1** in (C_5D_5N, 400 MHz)

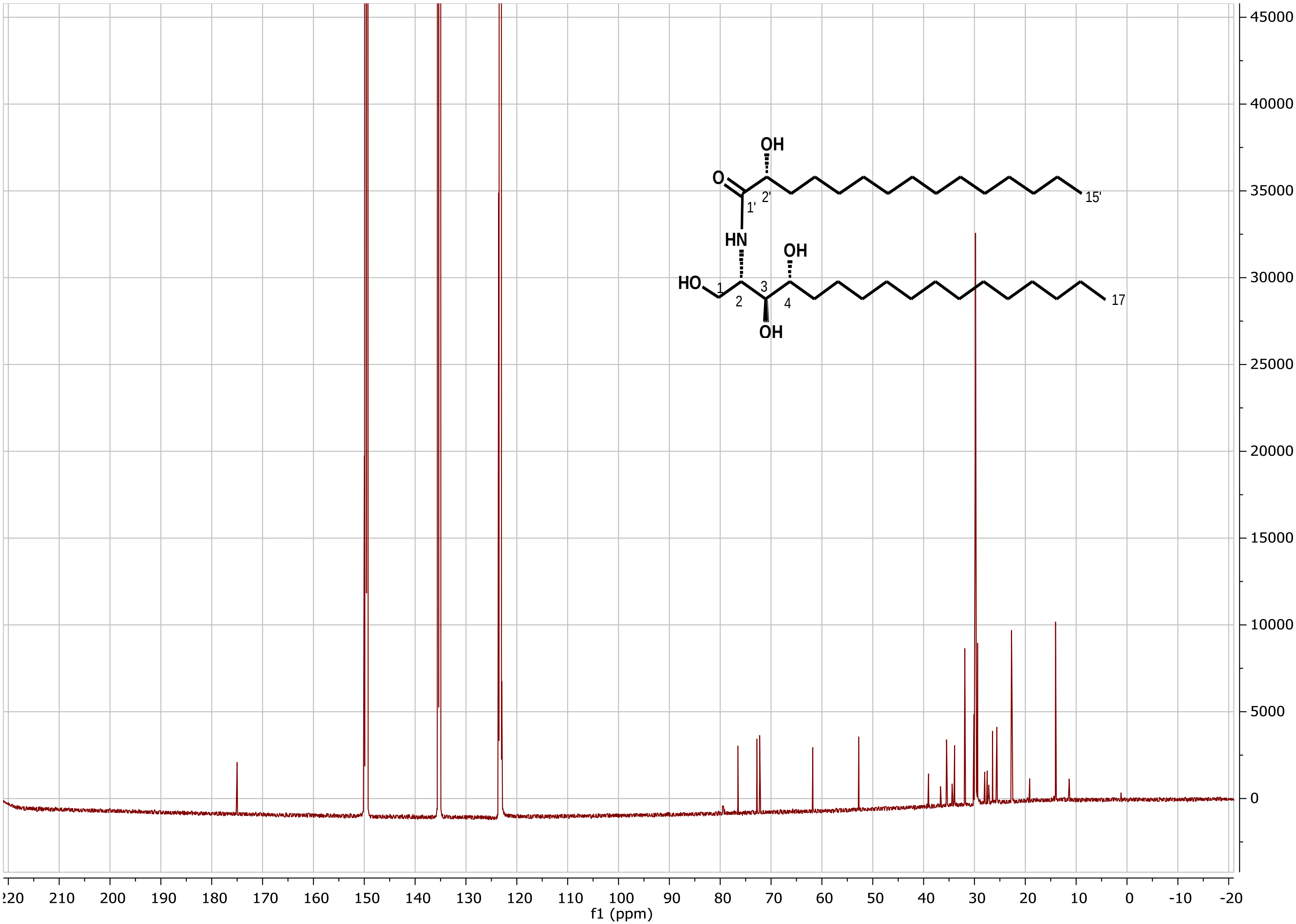

Figure S5: ^{13}C-NMR spectrum of compound **1** in (C_5D_5N, 100 MHz)

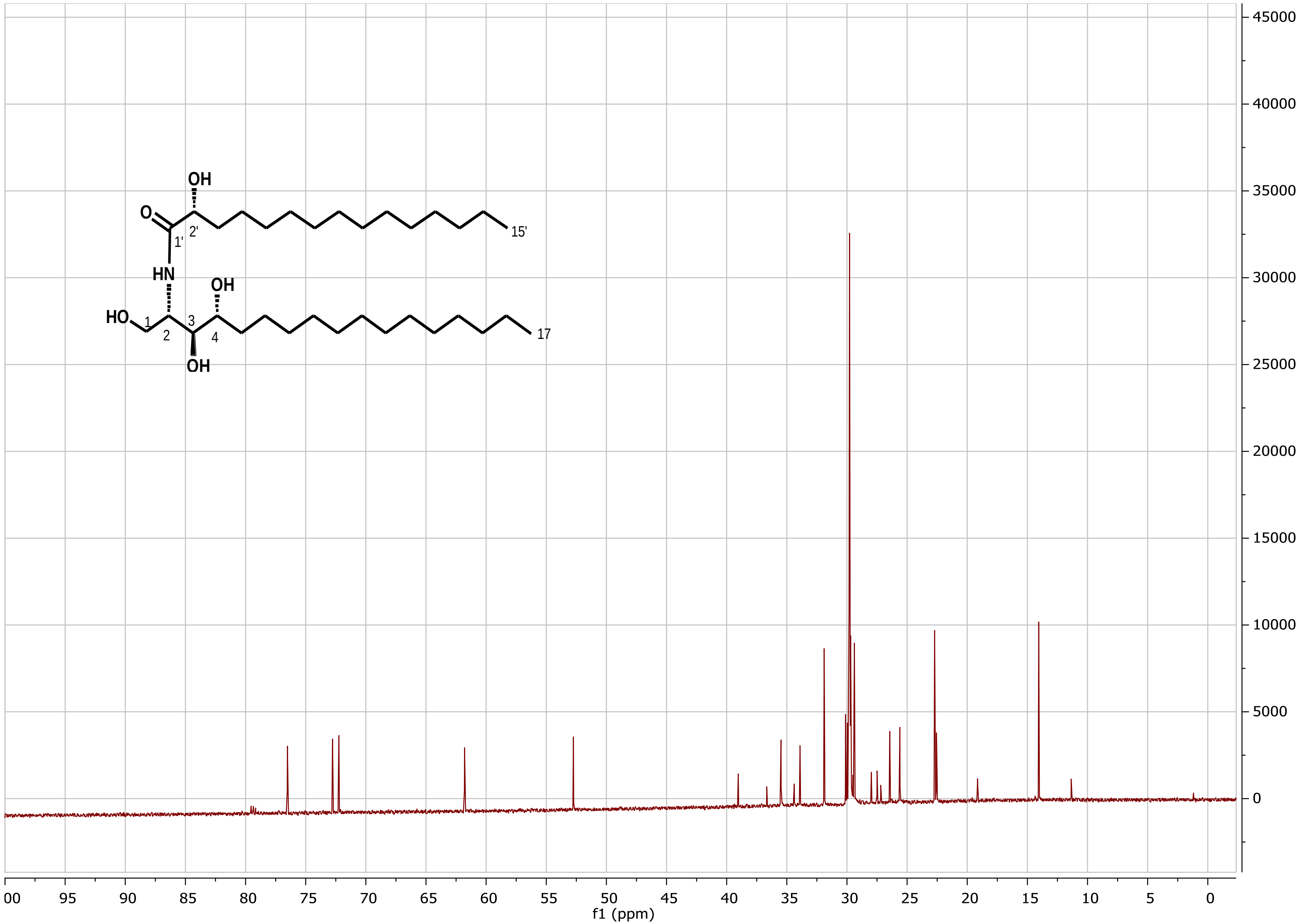

Figure S6: Partial expansion of the ^{13}C-NMR spectrum of compound **1** in (C_5D_5N, 100 MHz)

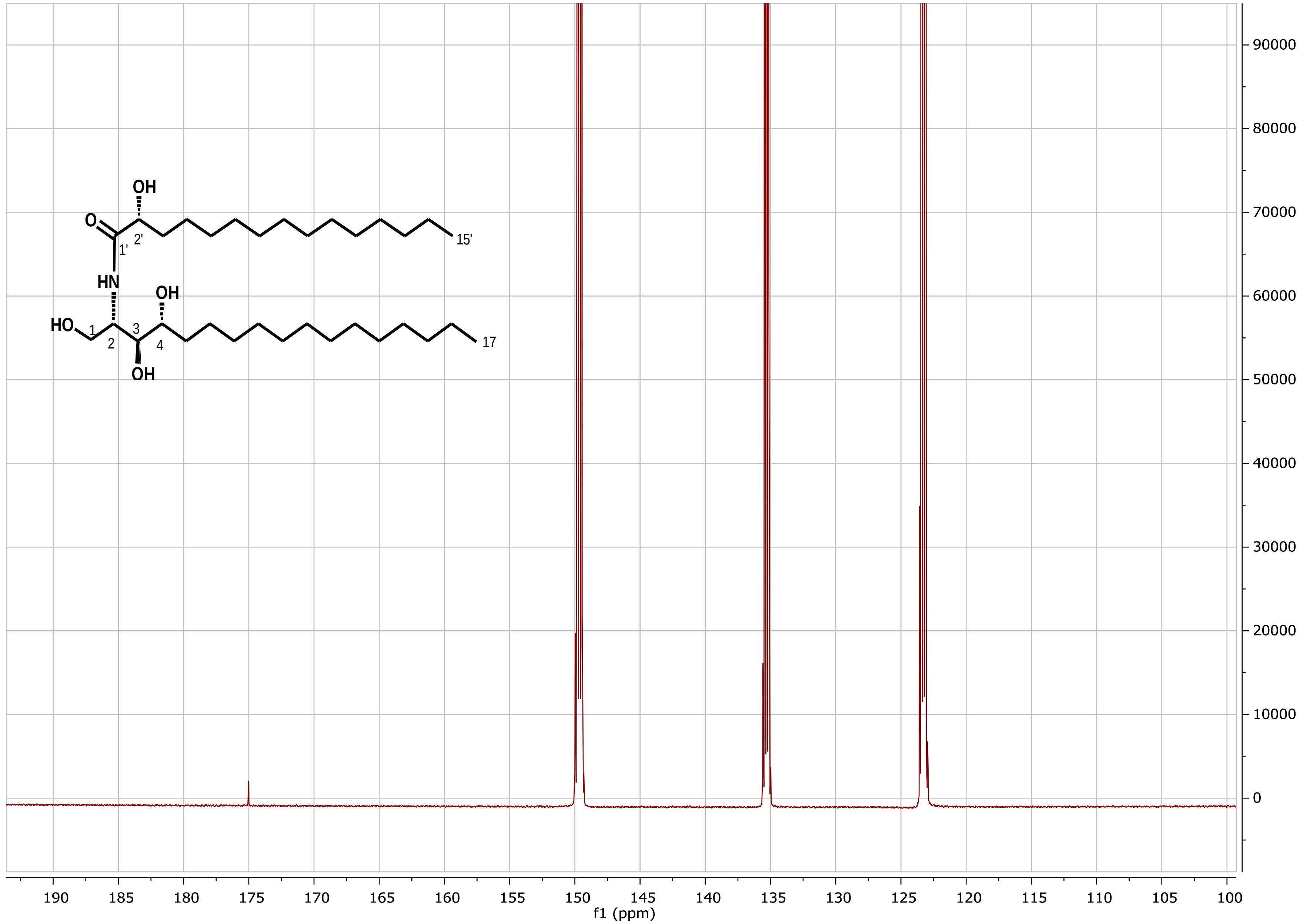

Figure S7: Partial expansion of the ^{13}C-NMR spectrum of compound **1** in (C_5D_5N, 100 MHz)

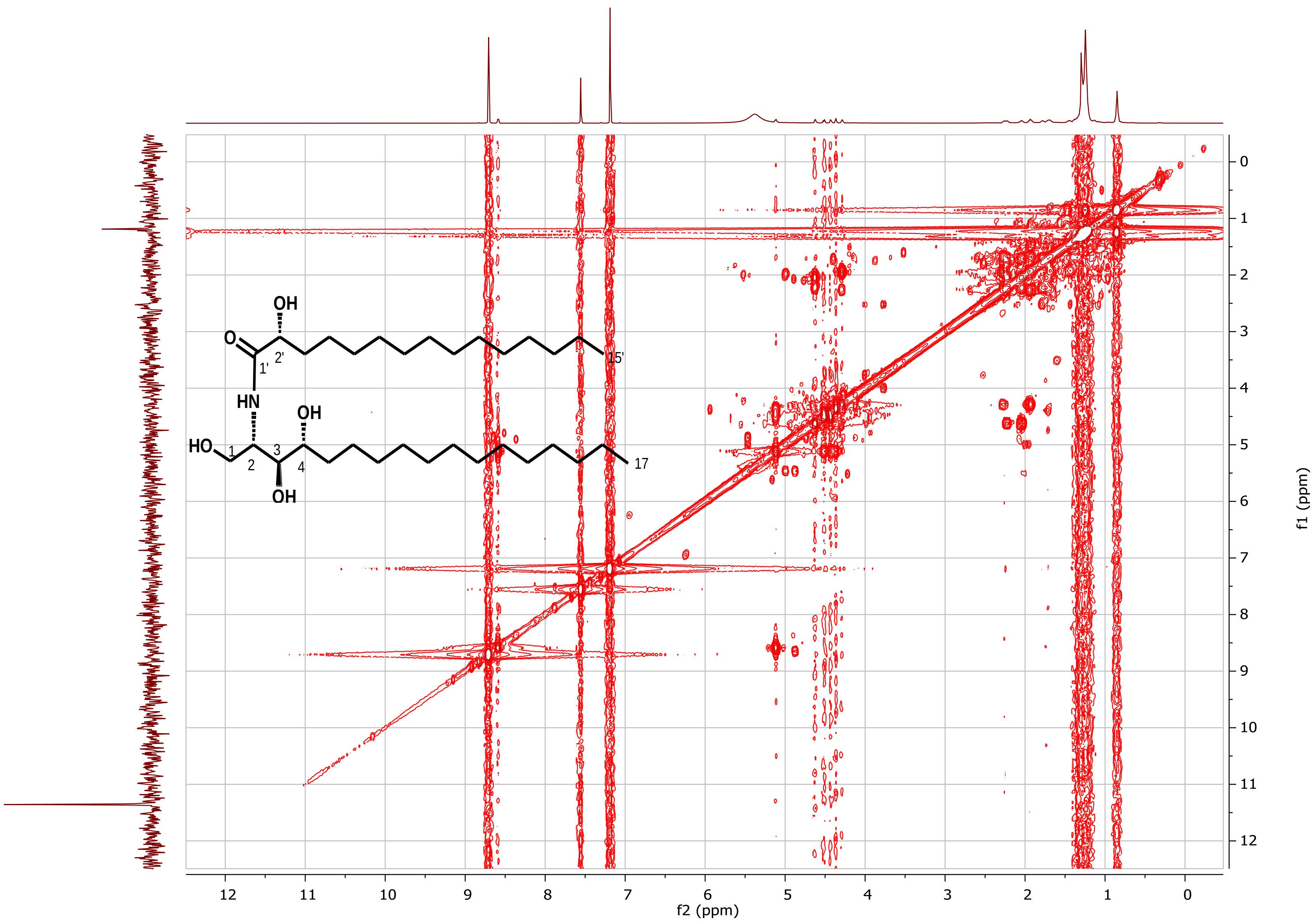

Figure S8: 2D-COSY spectrum of compound **1** in (C_5D_5N, 400 MHz)

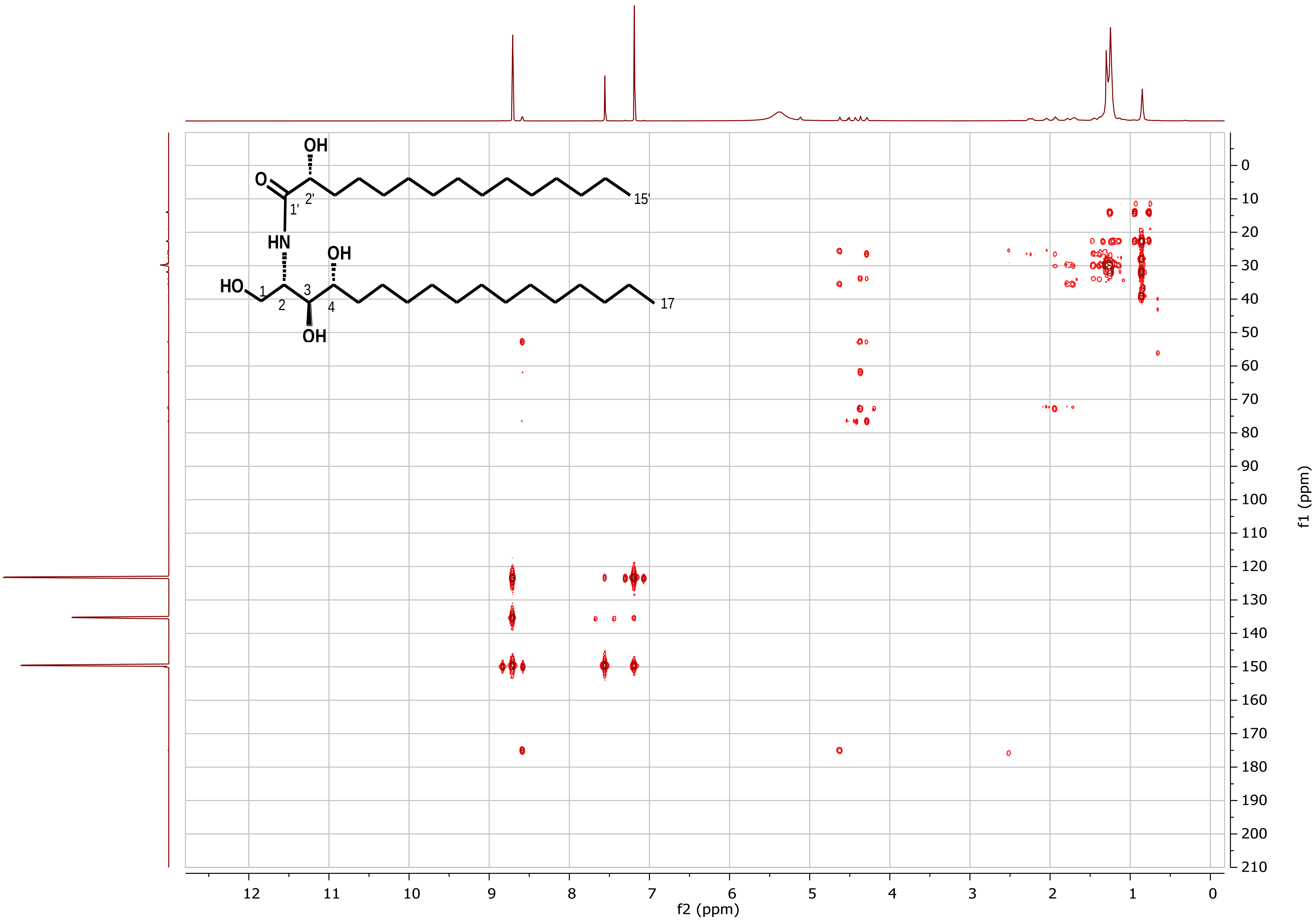

Figure S9: 2D-HMQC spectrum of compound **1** in (C_5D_5N, 400 MHz)

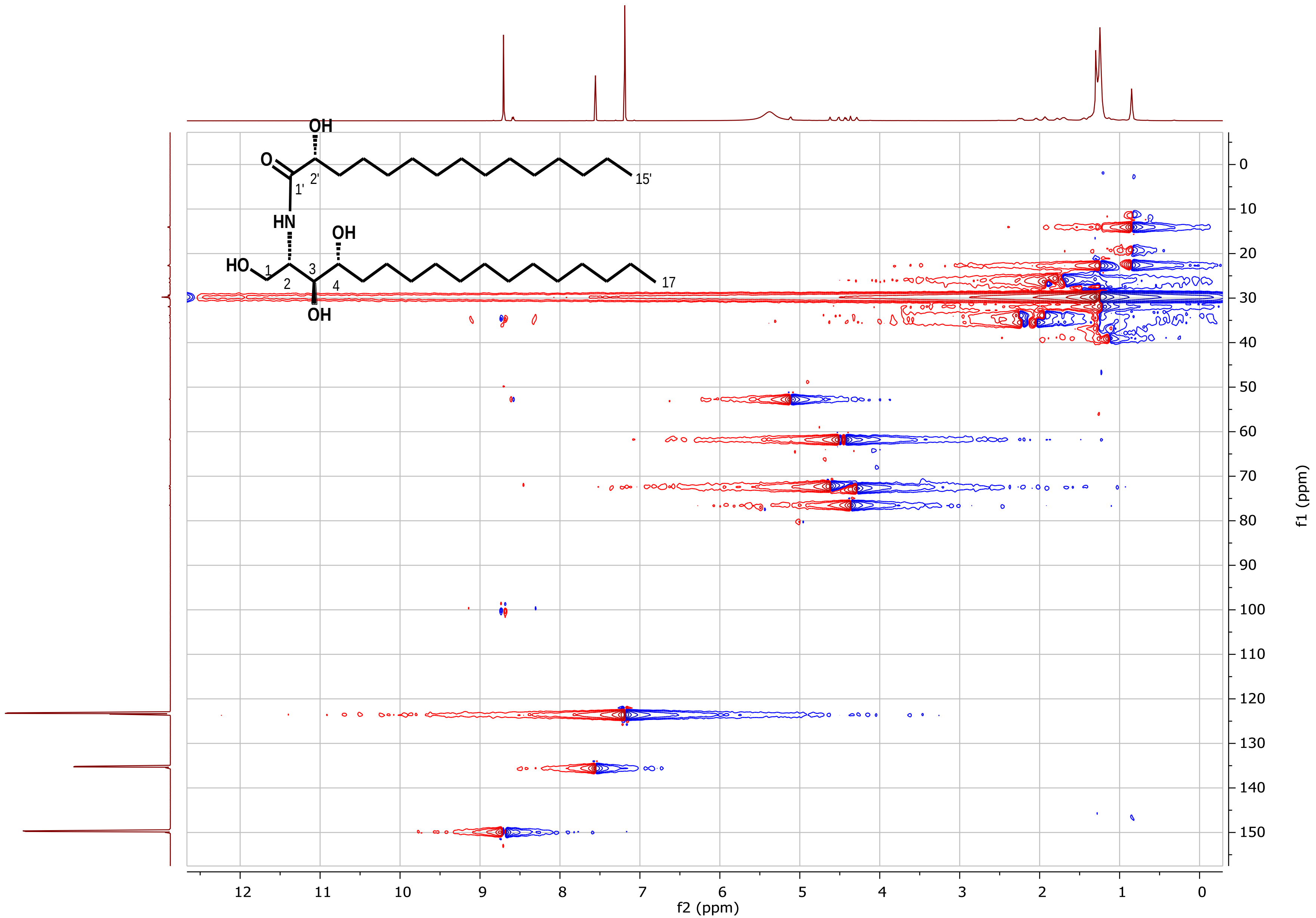

Figure S10: 2D-HMBC spectrum of compound **1** in (C_5D_5N, 400 MHz)

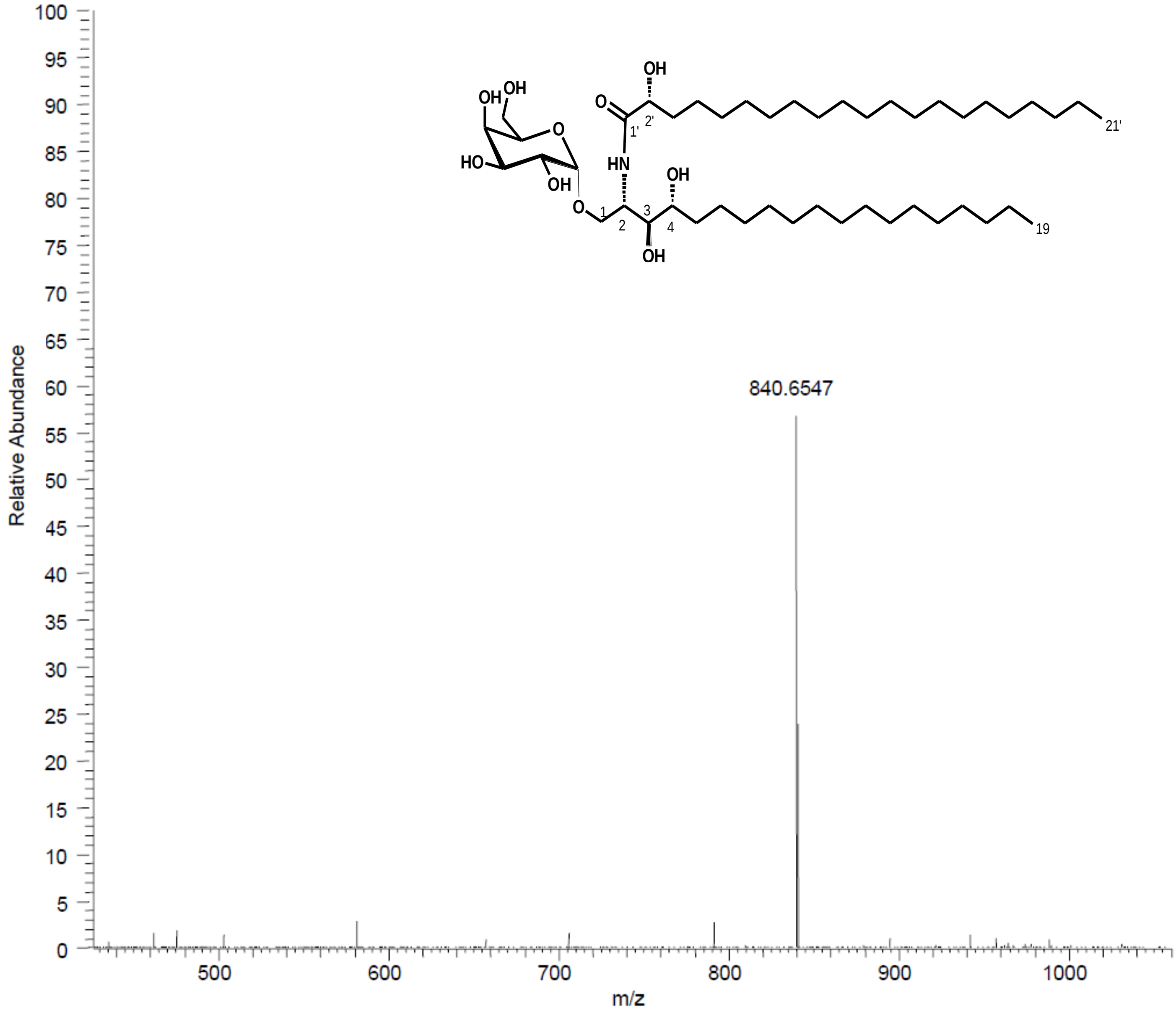

Figure S11: HRMS of Compound **2** (+MS**)**

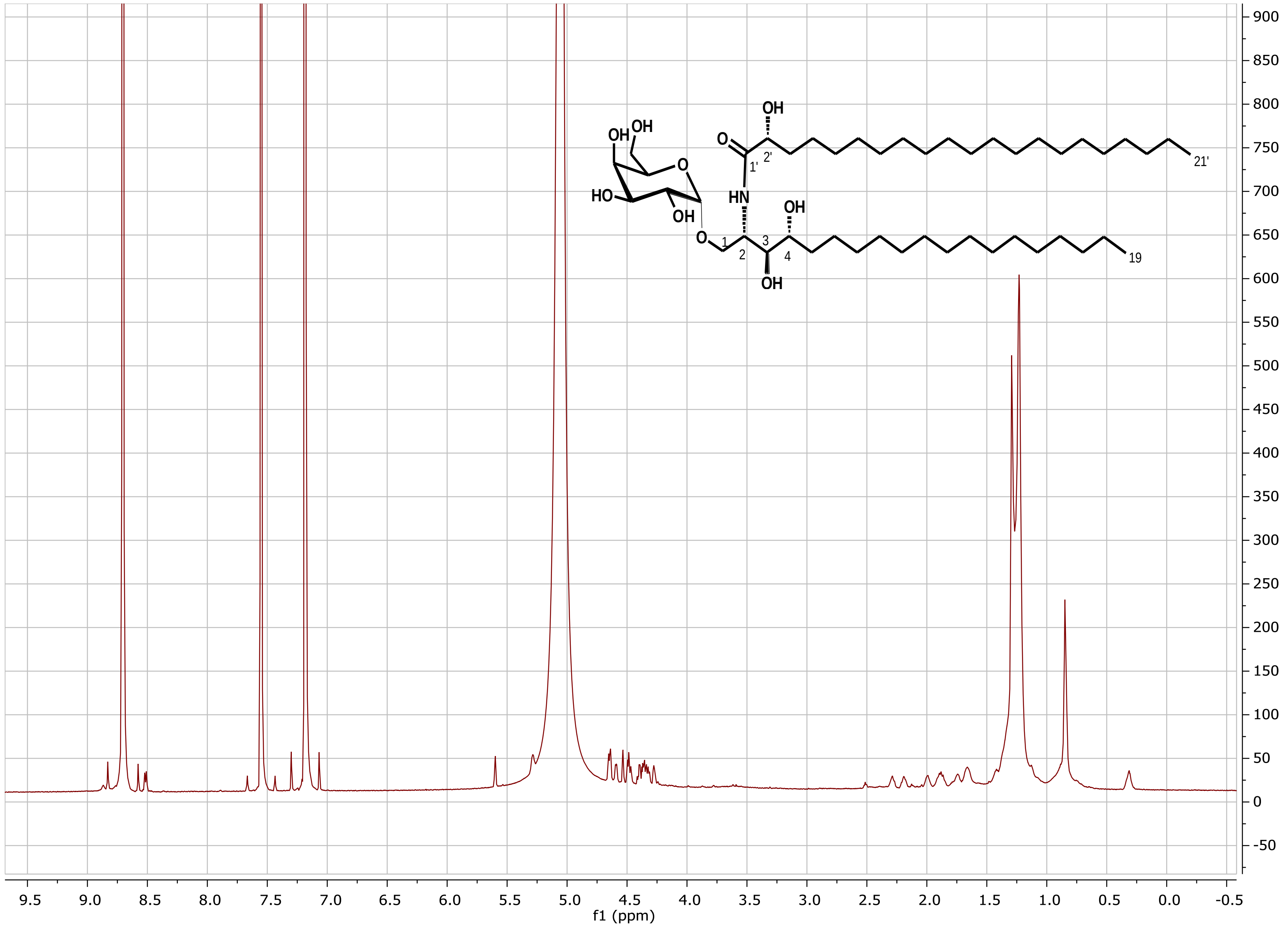

Figure S12: ^{1}H-NMR spectrum of compound **2** in (C_5D_5N, 400 MHz)

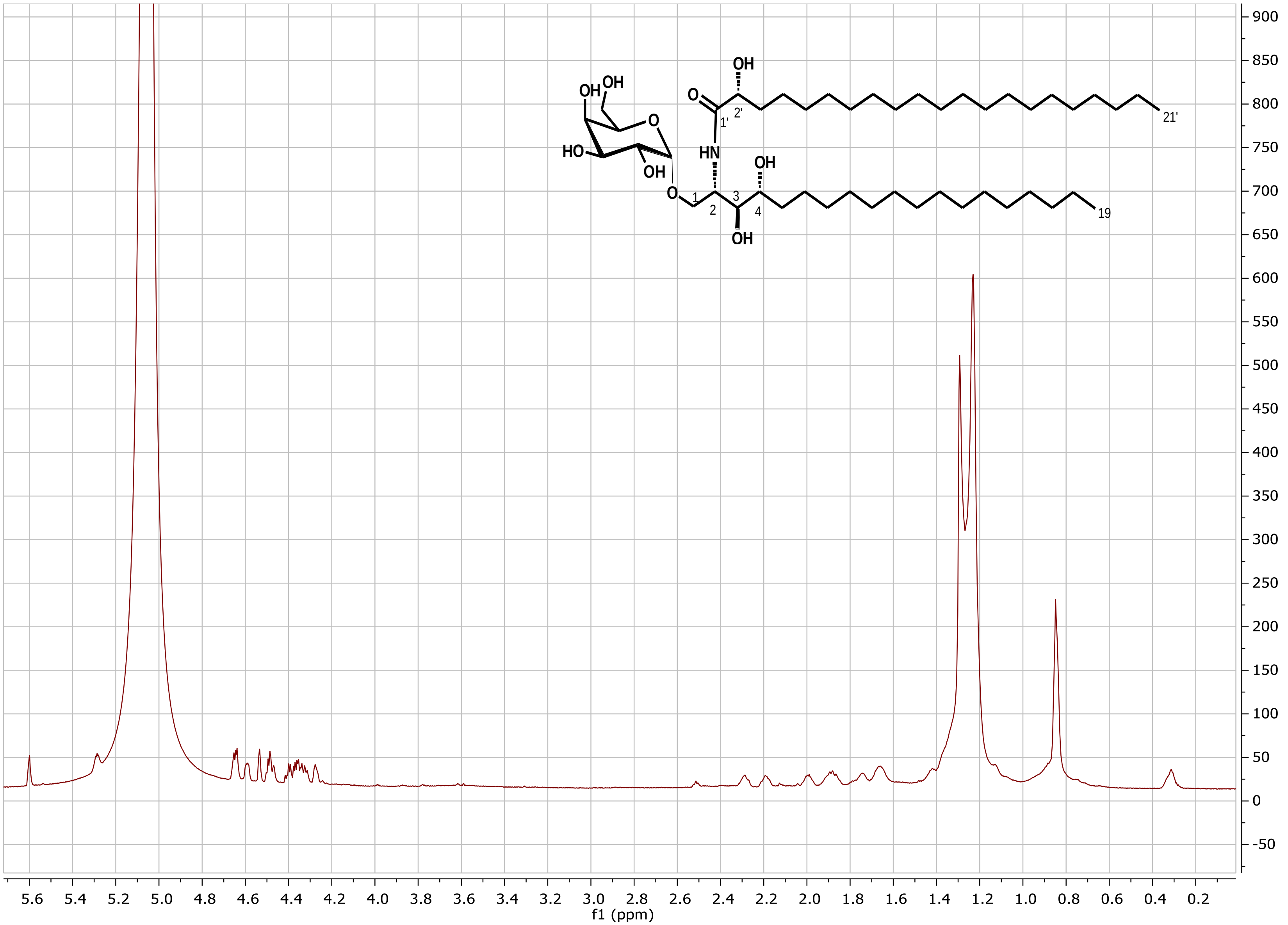

Figure S13: Partial expansions of the ^{1}H-NMR spectrum of compound **2** in (C_5D_5N, 400 MHz)

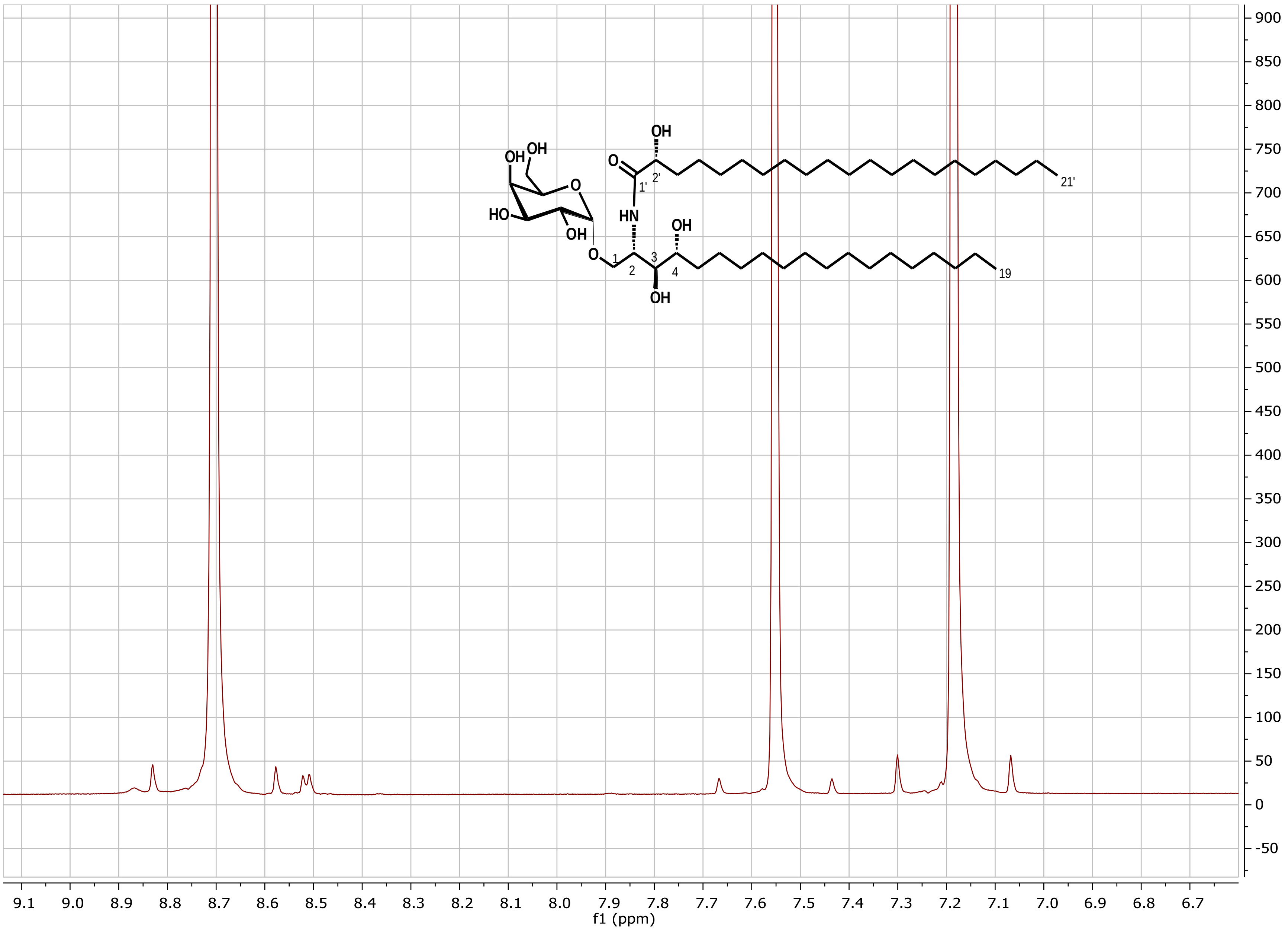

Figure S14: Partial expansions of the ^{1}H-NMR spectrum of compound **2** in (C_5D_5N, 400 MHz)

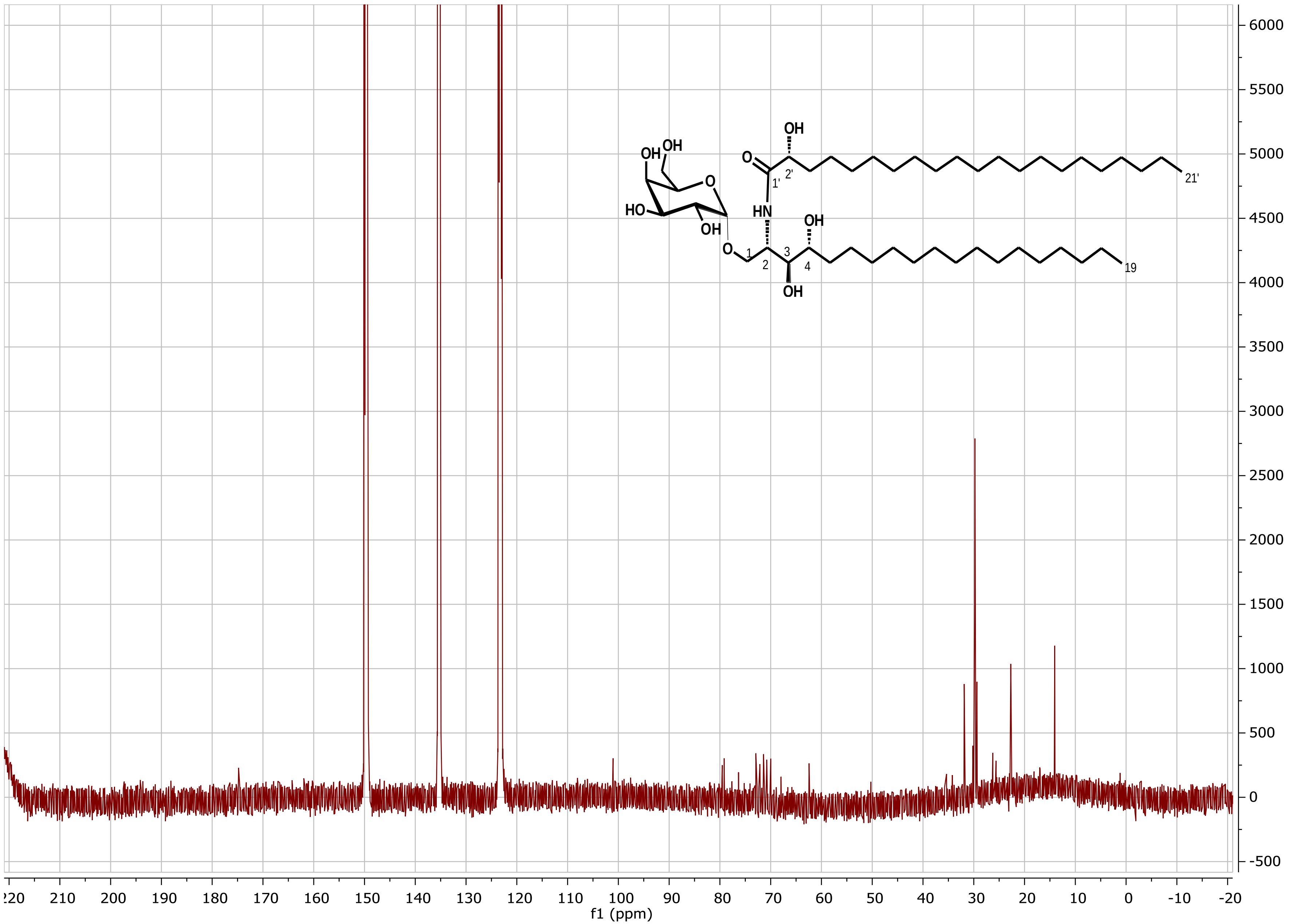

Figure S15: ^{13}C-NMR spectrum of compound **2** in (C_5D_5N, 100 MHz)

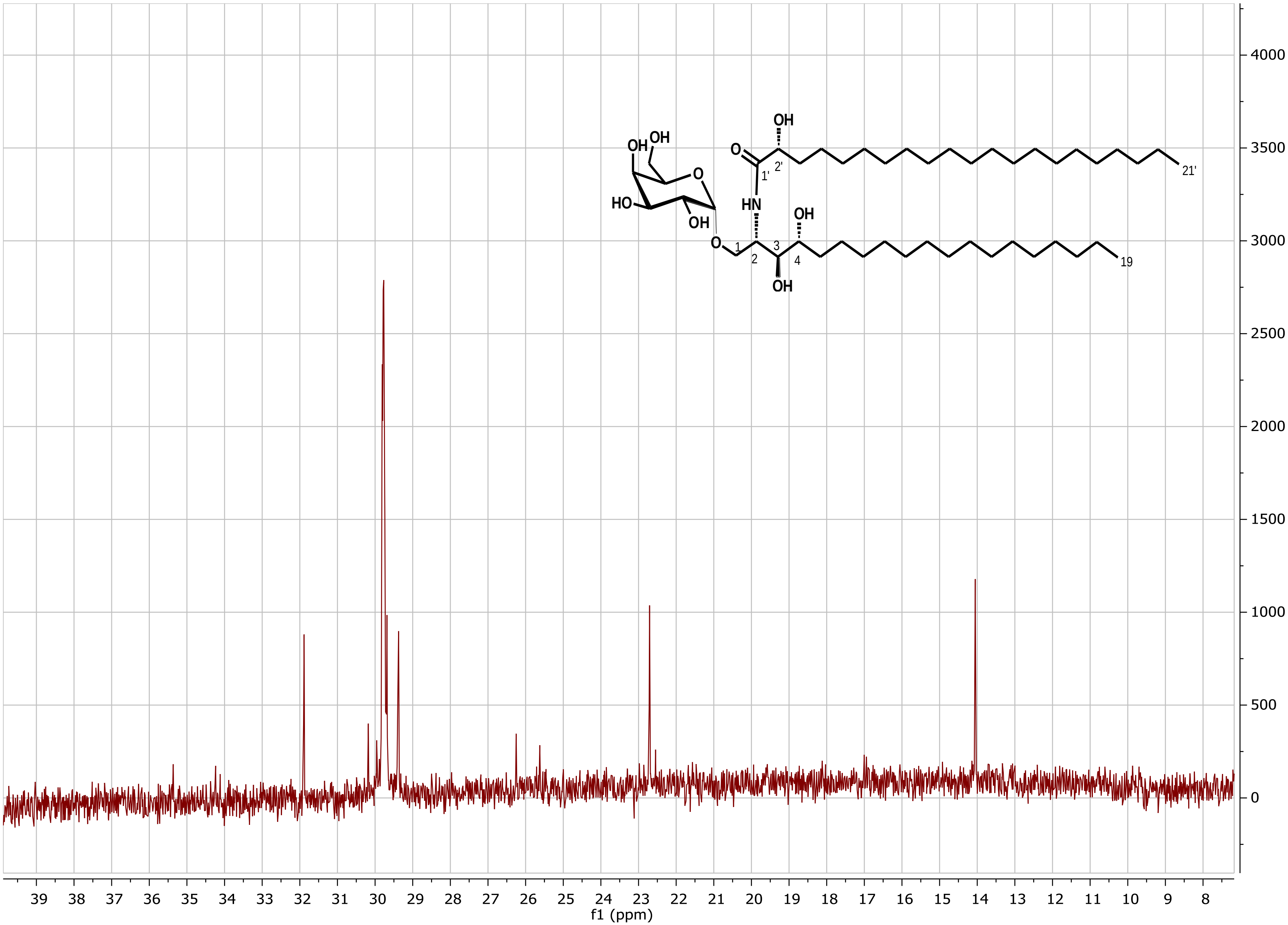

Figure S16: Partial expansion of the ^{13}C-NMR spectrum of compound **2** in (C_5D_5N, 100 MHz)

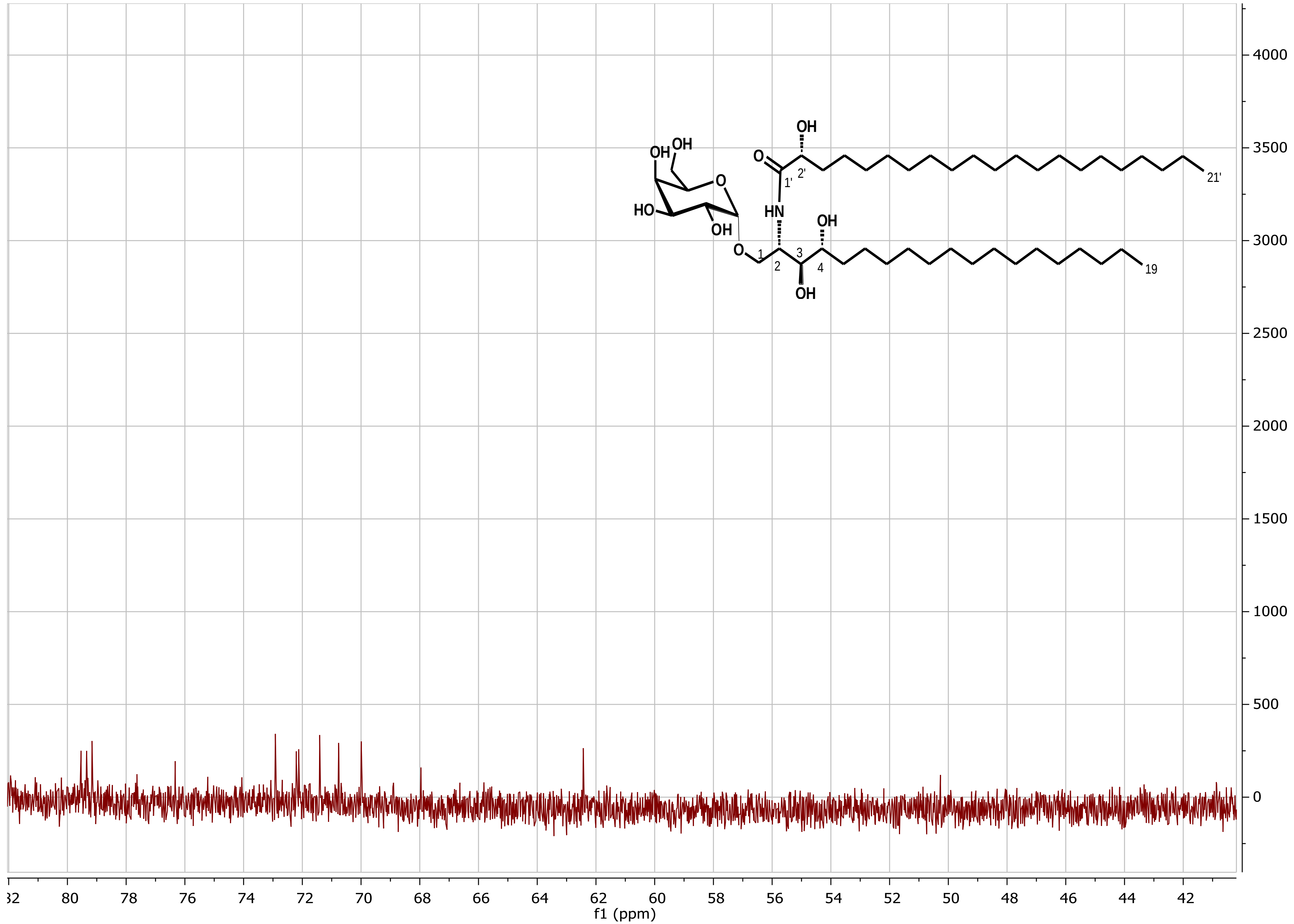

Figure S17: Partial expansion of the ^{13}C-NMR spectrum of compound **2** in (C_5D_5N, 100 MHz)

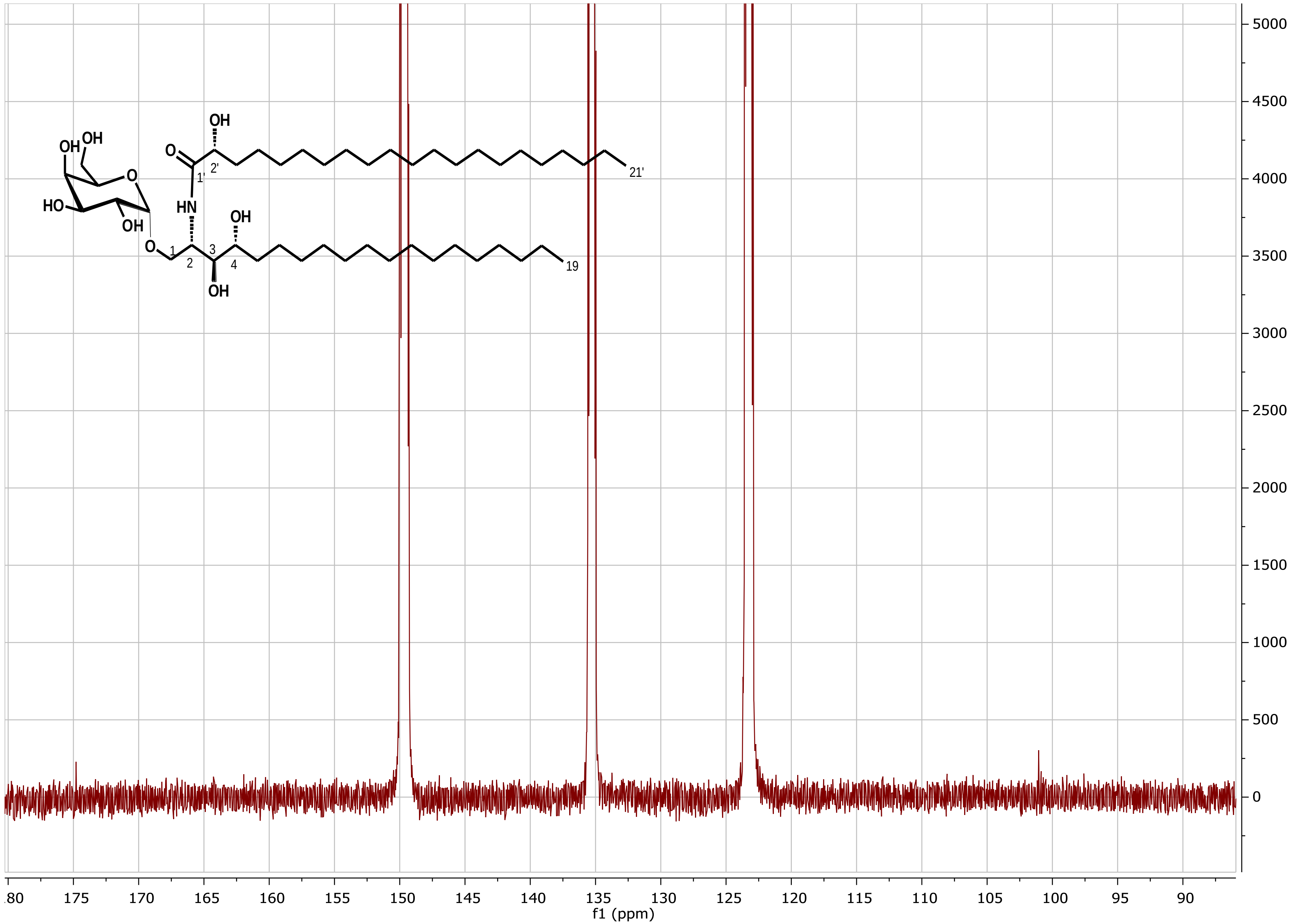

Figure S18: Partial expansion of the ^{13}C-NMR spectrum of compound **2** in (C_5D_5N, 100 MHz)

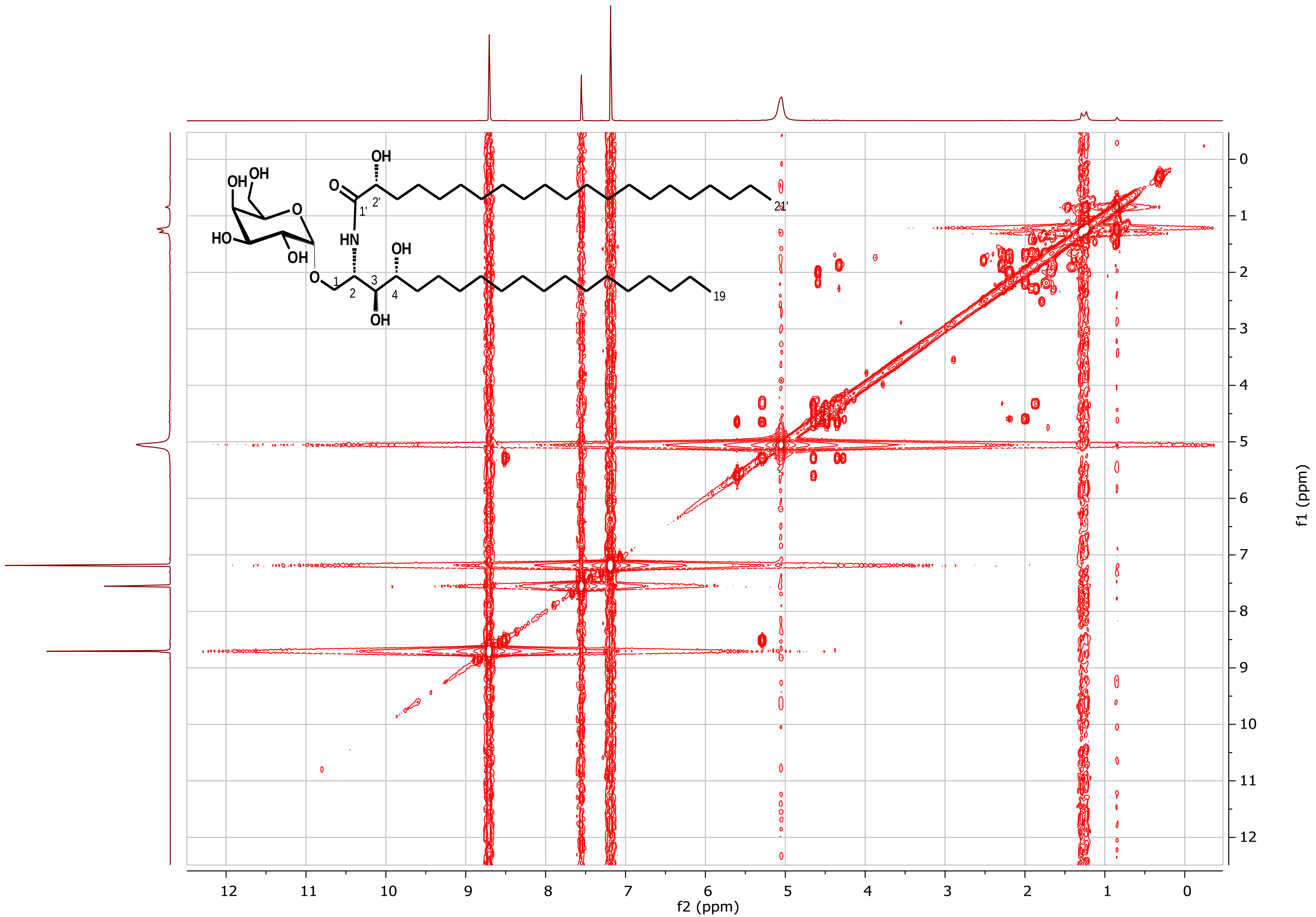

Figure S19: 2D-COSY spectrum of compound **2** in (C_5D_5N, 400 MHz)

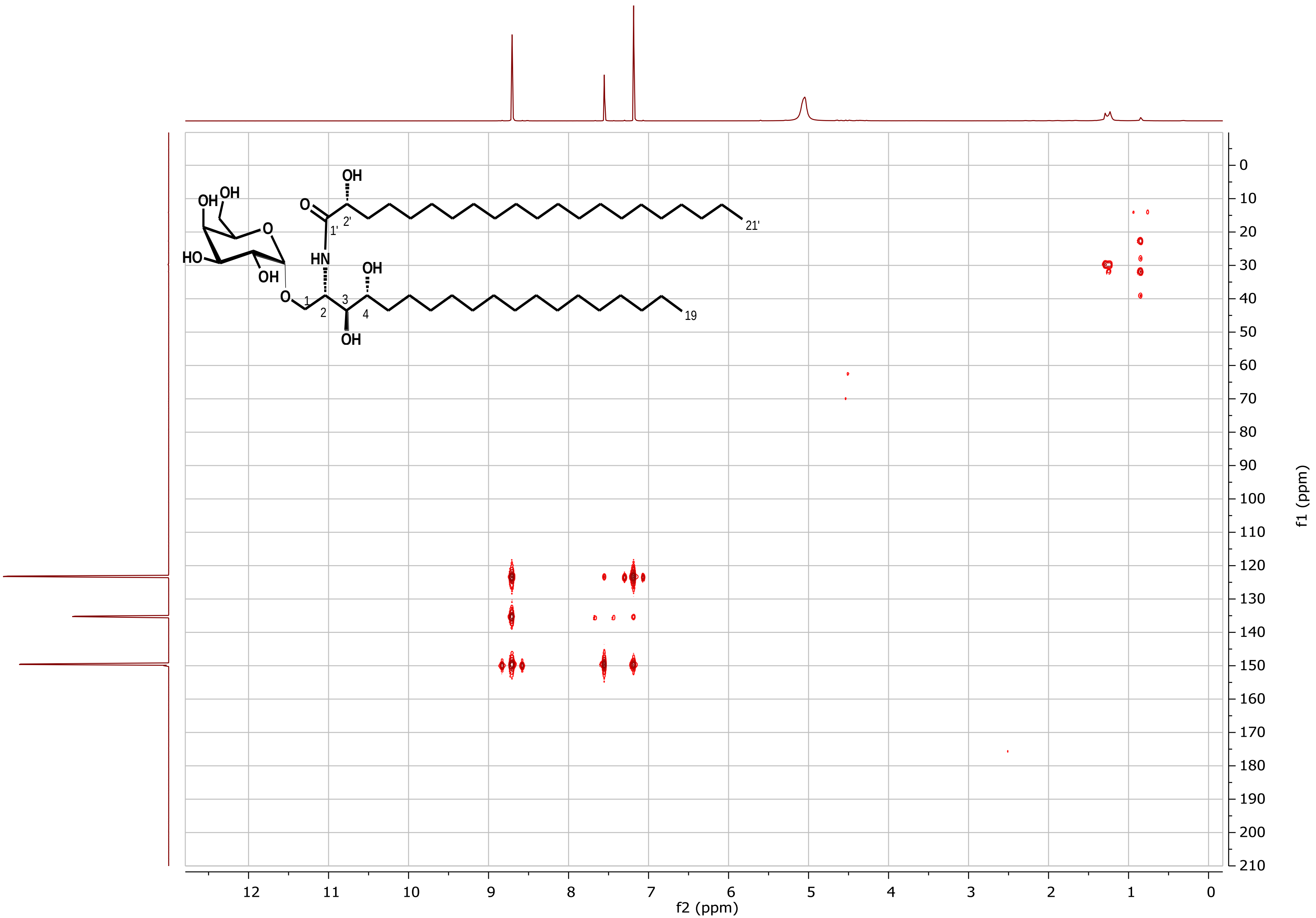

Figure S20: 2D-HMQC spectrum of compound **2** in (C_5D_5N, 400 MHz)

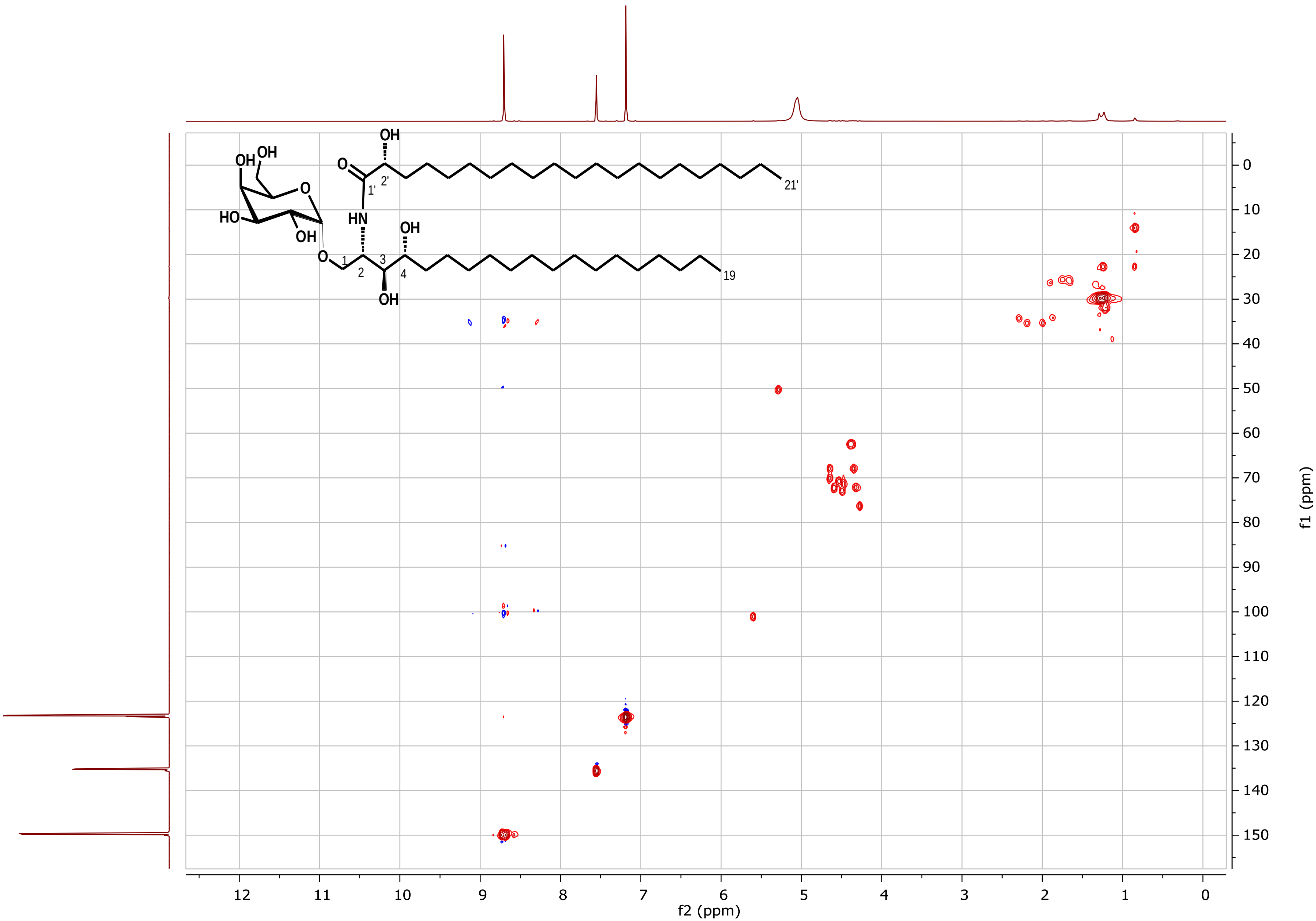

Figure S21: 2D-HMBC spectrum of compound **2** in (C_5D_5N, 400 MHz)
